# Solid-state fermentation by *Aspergillus niger* and *Trichoderma koningii* improves the quality of tea dregs for use as feed additives

**DOI:** 10.1371/journal.pone.0260045

**Published:** 2021-11-12

**Authors:** Yiyan Cui, Jiazhou Li, Dun Deng, Huijie Lu, Zhimei Tian, Zhichang Liu, Xianyong Ma

**Affiliations:** 1 Institute of Animal Science, Guangdong Academy of Agricultural Sciences, Guangzhou, China; 2 State Key Laboratory of Livestock and Poultry Breeding, Guangzhou, China; 3 The Key Laboratory of Animal Nutrition and Feed Science in South China, Ministry of Agriculture, Guangzhou, China; 4 Guangdong Provincial Key Laboratory of Animal Breeding and Nutrition, Guangzhou, China; 5 Guangdong Engineering Technology Research Center of Animal Meat Quality and Safety Control and Evaluation, Guangzhou, China; 6 Maoming Branch, Guangdong Laboratory for Lingnan Modern Agriculture, Maoming, China; Institute for Biological Research, University of Belgrade, SERBIA

## Abstract

This study evaluated the ability of *Aspergillus niger* and *Trichoderma koningii* to improve the quality of tea dregs (TDs) through solid-state fermentation as well as the value of the fermented tea dregs (FTDs) produced for use as bio-feed additives. After fermentation, FTDs differed in color and structure. Fermentation with *A*. *niger* and *T*. *koningii* increased the contents of crude protein, crude fiber, neutral detergent fiber, and acid detergent fiber of TDs. Compared to the unfermented group, the contents of reducing sugar, total flavonoids, total polyphenols, and theasaponins were increased in *A*. *niger* FTDs, while in *T*. *koningii* FTDs caffeine was completely degraded, the theasaponins were lower, and the contents of reducing sugar and caffeine higher. Regarding free amino acids, *A*. *niger* FTDs had the highest content of total amino acids, total essential amino acids, total non-essential amino acids, total aromatic amino acids, total branched-chain amino acids, and total non-protein amino acids, and all types of essential amino acids, followed by *T*. *koningii* FTDs and the control TDs. Fungal fermentation had similar effects on the content of various hydrolytic amino acids as those on above free amino acids, and increased the content of bitter and umami components. The composition of essential amino acids of TDs or FTDs was similar to that of the standard model, except for sulfur-containing amino acids and isoleucine. Solid-state fermentation with *A*. *niger* and *T*. *koningii* effectively improved the nutritional value of TDs, increased the contents of functional substances, and improved the flavor of TDs. This study demonstrated a feasible approach to utilize TDs that not only increases animal feed resources, but also reduces the production of resource waste and pollution.

## Introduction

Tea is a traditional beverage in China, and its beverage products are popular worldwide. In 2019, global tea production was approximately 6.5 million tons [1]. Tea dregs (TDs) are low-cost residues produced after the extraction of water-soluble substances and polyphenols from tea. In China, approximately 160,000 tons of TDs are discarded per year [2]. To date, the studies on TDs have focused on organic fertilizer [3], mushroom matrix [4], heavy metal adsorption, wastewater treatment [5], brick preparation [6], but there are few studies on the utilization of TDs for feed. This renewable biomass resource puts great pressure on the environment and also waste bioactive components in tea, including many nutrients that are present in TDs. The main nutrients in dry TDs include crude protein (16.3−35.65%), ether extract (1−7.4%), and minerals [7, 8]. In addition, TDs are rich in functional substances, such as polyphenols, alkaloids, saponins, and amino acids [9, 10], which have biological activities, such as antioxidant, immunoregulatory, anti-obesity, and anti-diabetic activities [10, 11]. Animal studies have shown that besides improving animal digestion, meat quality, serum antioxidant capacity, and intestinal morphology [12, 13], TDs also improve immunity and regulate the proportion of different types of muscle fibers [14]. Therefore, TDs are a promising source of feed additives. However, they contain some non-nutritional factors, such as saponins and caffeine, which are bitter in taste and low in palatability. Studies have shown that the amount of added TDs in animal feed is very low (0.5−2%), even in ruminant feed [15–18]. The amount of waste TDs generated is much larger than the amount used, which is not conducive to the efficient use of TDs. It is very important for the sustainable development of tea resources to select green and effective biotechnology to improve TDs, nutritional value, flavor, and palatability, as well as reduce the content of anti-nutritional factors and promote the utilization of TDs in animal feed.

Solid-state fermentation (SSF) is an easy to scale up, simple to operate, and a low-cost process, which can be used to produce bioactive substances that are beneficial to human health and improve the nutritional value of by-products. In general, SSF is an economically efficient technology for producing biologically active products. The hyphae of fungi spread over the surface of materials and easily penetrate the space between particles [19] and are characterized by rapid growth and high metabolic activity [20]. *Aspergillus niger* and *Trichoderma koningii* are inherently safe and reliable. Fermentation with *A*.*niger* can produce abundant enzymes, which contribute to the degradation of macromolecular substrates, the transformation of bioactive components, and the synthesis of new compounds, and ultimately improve the nutritional and functional properties of substrates [21]. In one study, the content of crude protein in TDs fermented by *A*. *niger* was increased [22], but the contents of flavonoids, polyphenols, reducing sugars and other active substances were not determined. It is not clear whether *A*. *niger* increases the content of active substances. *T*. *koningii* is often used in the biological control of plant pathogens. It can reproduce using a variety of carbon or nitrogen sources under adverse environmental conditions [20], and is a dominant strain for decomposing sorghum straw crude fiber [23]. After *T*. *koningii* fermentation, the crude protein content of mulberry leaves was 31.27%, and the protease production activity was 107.17 U/mL [24]. However, to the best of our knowledge, there is no research on the fermentation of TDs by *T*. *koningii*. It is unclear whether *T*. *koningii* improves the nutritional components, active substances, anti-nutritional factors, etc. of TDs. In addition, the high fiber content and bitter and astringent taste of TDs lead to less consumption of TDs by animals. Fungi secrete abundant enzymes, such as proteases and cellulases, which decompose macromolecular substances into small molecular substances, such as glucose and amino acids, thereby improving the flavor of feed. The contents of free amino acids fermented tea leaves of *Grifola frondosa* and *Mesona chinensis* were 1.52 and 0.94%, respectively, and the types of amino acids increased significantly to improve the flavor of tea leaves [9]. However, to date, there has been no relevant research on the flavor of TDs fermented by *A*.*niger* or *T*. *koningii*, and it is unclear whether fungi can improve the flavor of TDs.

Here, we used SSF by fungi to produce fermented tea dregs (FTDs). The increase in the contents of crude protein, ash, minerals, active substances, and hydrolyzed amino acids in the fermented product demonstrated that fungal fermentation improved the nutritional value of TDs, and the evaluation of the content of free amino acids and flavor components showed that the palatability of FTDs was changed. The improvement of the nutritional value and palatability indicates that increasing the addition of TDs to animal feed could contribute to increase animal production. The use of FTDs not only reduces the impact of TDs on the environment but can also be a feed supplement for livestock and poultry and reduce costs, making FTDs an eco-friendly and economically effective approach to achieve a circular economy and turning agro-industrial by-products into capital.

## Materials and methods

### Materials

#### Tea dregs

TDs were collected from Kangshifu Beverage Co., Ltd. (Guangzhou,China). TDs were dried at 60°C through commercial production procedures. The dried TDs were ground into powder and screened through 40 mesh sieves. The contents of crude protein, ether extract, crude fiber, neutral detergent fiber (NDF), acid detergent fiber (ADF), ash, and carbohydrates in the TDs were 259, 27, 255, 455, 273, 388, and 676 g/kg, respectively. The contents of calcium, phosphorus, potassium, magnesium, manganese, iron, zinc, and copper were 4961, 7389, 4090, 1670, 986, 455, 28, and 12 mg/kg, respectively.

#### Strains

*T*. *koningii* and *A*. *niger* were isolated from fungal isolates obtained from *Pericarpium citri reticulatae* and identified by 18S rDNA molecular analysis and sequencing [25]. *A*. *niger* was identified as strain APBSDSF57 (MG669189.1), and *T*. *koningii* was identified as strain CTCCSJ-G-HB40448 (KY764915.1). These strains did not produce aflatoxin B1, T-2 toxin, zearalenone, deoxynivalenol, ochratoxin in FTDs (S1 File).

### Fermentation preparation

Potato dextrose agar medium (PDA), obtained from Huankai Microbial Science and Technology Co., Ltd. (Guangzhou, China) was used as an cultivation medium. Briefly, 0.2 mL of 2−3×10^8^ spores/mL conidium suspension was applied to the agar surface in a 10-cm Petri dish. To prepare the conidia suspension, the surface of the medium covered with fungi was first scraped with a sterile blade. The fungi and culture medium were then transferred to 15-mL centrifuge tube along with 5 mL of sterile water, followed by homogenization on a vortex mixer for 15 min. Eventually, a 10-mL syringe with 1.5-cm high absorbent cotton was used to filter the suspension twice to obtain the spore suspension. The concentration of spores was determined using a counting chamber. In the experiment, TDs and cornmeal (8:2 ratio) were mixed and used as a fermentation substrate. The matrix and water were mixed in a mass ratio of 4:6. The 40-g mixture was placed into a 200-mL bottle, which was then covered with a hydrophobic fluorine-perforated membrane, and the culture was sterilized at 121°C for 20 min. Subsequently, each bottle was inoculated with 1.6 mL of spore suspension (1.0×10^7^ spores/mL), and ten copies of each fungal strain were made. The spore suspension and the sterilized substrate were stirred more than 50 times using a sterile glass rod under strictly aseptic conditions, and then cultured at 28°C for 14 d. After fermentation, the inoculated samples were freeze dried by using an ALPHA 2–4 LSC freeze-dryer (Martin Christ GmbH, Osterode am Harz, Germany) for 48 h for analysis. After freeze-drying, the characteristics (color, structure, and odor) of the fermentation products were recorded. Eight expert panel members evaluated the odor of fermentation products according to S1 Table.

### Proximate component analysis

Proximate component analysis was performed according to Yan et al. [26]. The dry matter (DM) content of TDs was analyzed by drying the samples in an ALPHA 2–4 LSC freeze-dryer (Christ Martin GmbH) for 72 h. The crude protein content of TDs was analyzed by the Kjeldahl method. The catalyst and concentrated H_2_SO_4_ were added and digestion was performed at 420°C for 2 h, and nitrogen was determined by using a Foss™ Kjeltec™ 8400 Automatic Nitrogen Analyzer (FOSS, Hillerod, Denmark). The Soxhlet extraction method was used to measure the concentration of ether extract. Samples of 2 g were placed in petroleum ether to perform the extraction process for 2 h on a Foss™ 2055 SOXTEC Fat Analyzer (FOSS). After extraction, the weight of ether extract was measured and the concentration of ether extract was calculated relative to the crude fat weight. The crude fiber, NDF, and ADF contents were determined using fiber filter bags and an Ankom 2000 Fiber Analyzer (Ankom Technology Corporation, Macedon, NY, USA) following the procedure of Van Soest et al. [27]. The NDF content was assayed using sodium sulfite and a heat stable amylase and expressed inclusive of residual ash. The ADF content was expressed inclusive of residual ash. Ash was measured in a 1-g of sample that was carbonized to smokeless, and then burned in a SX2-4-10N Box Resistance Furnace (Yiheng, Shanghai, China) at 550°C for 6 h. After cooling, the sample was weighed. The carbohydrate content was calculated by subtracting the contents of crude protein, ether extract, and ash from 1 kg of DM and expressed as g/kg DM.

### Mineral analysis

The phosphorus content was determined by ammonium vanadium molybdate spectrophotometry at 400 nm using a Spectra Max^®^ M5Multi-modeMicroplate Reader (Molecular Devices LLC., San Jose, CA, USA).The contents of calcium, copper, iron, magnesium, manganese, potassium, sodium, and zinc in the samples were determined according to GB5009.268–2016 [28]. The sample (0.5 g) was placed in the microwave digestion inner tank and diluted to 10 mL with 68%nitric acid, and microwave digested for 1h. After microwave digestion and cooling, the sample was ultrasonically degassed for 5min, and the contents were diluted to 50 mL with ultrapure water. The signal response values of the elements to be measured and the internal standard elements were measured by injecting the sample solution into the ICP-MS 7700X, Inductively Coupled Plasma Mass Spectrometer (Agilent Technologies Inc., Santa Clara, CA, USA).The concentration of the elements to be measured in the digestion solution was determined according to the standard curve, and the content was expressed as mg/kg DM.

### Feeding value analysis

The feeding value index was calculated according to Xiong et al. [29]. Total digestible nutrient (TDN, %DM) = 82.38–0.7515 × ADF (%DM), digestibility of dry matter (DDM, %DM) = 88.9–0.779 × ADF (%DM), dry matter intake (DMI, %BW) = 120/NDF (%DM), relative feed value (RFV) = DMI (%BW) × DDM (%DM)/1.29, and relative forage quality (RFQ) = TDN (%DM) × DMI (%BW)/1.23.

### Bioactive substance analysis

#### Reducing sugar content analysis

To measure the concentration of reducing sugar, 0.1-g samples were each dissolved in 10 mL of distilled water, mixed and incubated for 1 h at 50°C, shaking every 10 min. Afterwards, 2 mL of solution was removed and centrifuged at 4000 × *g* for 10 min. One mL of supernatant was mixed with 2 mL of dinitrosalicylic acid reagent and the mixture was boiled for 5 min. After cooling, 9 mL of distilled water was added to the mixture. Then, spectrophotometric measurements were performed on a Spectra Max^®^ M5 Multi-mode Microplate Reader (Molecular Devices LLC.) at 540 nm. The concentration of total reducing sugars was calculated based on a calibration curve of glucose.

#### Total flavonoid content analysis

Procedures for the extraction and determination of total flavonoids were as described by Wang et al. [30]. The samples (0.5 g) were each mixed with 10 mL of 80% (v/v) methanol and sonicated at 25 kHz and 100 W for 60 min, and then stored in the dark for 24 h with mixing every 8 h. After centrifuging the extract solution at 4000 × *g* for 10 min, 5 mL of the supernatant or the rutin standard solution was mixed with 0.3 mL of 5% NaNO₂ for 10 min. Then, 0.3 mL of 10% AlCl_3_ was added to the mixture and allowed to stand for 10 min. Afterwards, 2 mL of 1 mol/L NaOH and 2.4 mL of 80% methanol were added, and the mixture was left to stand for a further 10min. Eventually, the absorbance was measured at 415 nm on a Spectra Max® M5 Multi-mode Microplate Reader (Molecular Devices LLC.). The total flavonoid content was expressed as g rutin equivalent (RE)/100g DM.

### Total polyphenol content analysis

Total polyphenols content was determined by the Folin–Ciocalteu method. Samples (0.2 g) were mixed with 10 mL of 70% (v/v) methanol, and then stored at 70°C for 1 h with mixing every 10 min. Then, the extract solution was centrifuged at 4000 × *g* for 10 min, and1 mL of the extract was then mixed with 5 mL of Folin–Ciocalteu’s reagent. After 5 min, 4 mL of 7.5% NaCO_3_ was added to the mixture, which was then left to stand for 1 h at room temperature. Subsequently, the absorbance was measured at 765 nm on a Spectra Max® M5 Multi-mode Microplate Reader (Molecular Devices LLC.).The total polyphenol content was expressed as g gallic acid equivalent (GAE)/100g DM.

#### Theasaponin content analysis

Theasaponin content was determined by the sulfuric acid vanillin colorimetric method. Samples (0.1 g) in 0.9 mL of 60% (v/v) ethanol were extracted at 60°C for 3 h. After collecting the extract by centrifugation at 4000 × *g* for 10 min, 0.5 mL of extract or theasaponin standard solution was mixed with 4 mL of 77% (v/v) H_2_SO_4_ and 0.5 mL 8% (m/v) vanillin ethanol solution and stored at 60°C for 15 min. After cooling the extract in ice water for 10 min the absorbance was measured at 550 nm on a Spectra Max® M5 Multi-mode Microplate Reader (Molecular Devices LLC.). Theasaponin content was expressed as mg/100g DW.

#### Caffeine content analysis

The content of caffeine was determined by first immersing, samples of 0.1 g mixed with 0.45 g MgO in 30 mL of boiling water at 100°C for 1 h with shaking every 10 min. After cooling the samples, the volume of the sample solution was increased to 50 mL by adding distilled water, and the solution was filtered through a 0.45-μm filter. Caffeine was determined by high performance liquid chromatography using a Waters Alliance HPLC e2695 Separations Module (Waters Corporation, Milford, USA) equipped with an ultraviolet/visible2489 detector. The chromatographic conditions were as follows: UV detector 280 nm, 1 mL/min flow rate, 40°C column temperature, and 10 μL injection volume. Caffeine content was expressed as mg/100g DM.

### Antioxidant capacity analysis

To measure antioxidant capacity, TDs were first extracted with water, 70% ethanol, and 70% methanol. Samples (1 g) were extracted with solvent at a 1:9 (m:v) ratio, followed by sonication at 25 kHz and 100 W for 60 min. The extract was collected by centrifugation at 4000 × g for 10 min. Vitamin C (2 mg/mL) was used as the positive control. The radical scavenging ratio assay was performed according to the methods of Li et al. [31].

#### 2, 2-diphenyl-1-picrylhydrazyl (DPPH) assay

The extract (0.5 mL) and 2.5 mL of a DPPH solution (0.1 mmol/L) were mixed and incubated at 37°C for 1 h. The blank group was treated with distilled water instead of extract, and the control group was dissolved in 50% ethanol instead of DPPH. The absorbance was measured at 510 nm on a Spectra Max® M5 Multi-mode Microplate Reader (Molecular Devices LLC.).

The DPPH scavenging ratio was calculate using the following formula: DPPH scavenging ratio (%) = (A_blank_−(A_extract_− A_control_)) /A_blank_× 100

where A_blank_ is the absorbance with distilled water instead of extract, A_extract_ is the absorbance with extract, and A_control_ is the absorbance with 50% ethanol instead of DPPH.

#### 2, 2-azinobis-3-ethylben-zothiazoline-6-sulfonic acid diammonium salt (ABTS+) assay

The ABTS^+^ solution (0.8 mL) was mixed with 0.2 mL of extract and was allowed to stand for 10 min. The blank group was treated with distilled water instead of extract, and the control group was dissolved with distilled water instead of ABTS^+^ solution. The absorbance was measured at 734 nm on a Spectra Max® M5 Multi-mode Microplate Reader (Molecular Devices LLC.).

The ABTS^+^ scavenging ratio was calculate using the following formula: ABTS^+^ scavenging ratio (%) = (A_blank_− (A_extract_− A_control_)) /A_blank_× 100

where A_blank_ is the absorbance with distilled water instead of extract, A_extract_ is the absorbance with extract, and A_control_ is the absorbance with distilled water instead of the ABTS^+^ solution.

#### OH scavenging assay

The extract (0.5 mL), 1 mL FeSO_4_ (2 mmol/L), 1 mL H_2_O_2_ (8 mmol/L) and 0.5 mL salicylic acid (6 mmol/L) were mixed and incubated at 37°C for 1 h. The blank group was treated with water instead of extract, and the control group was dissolved in distilled water instead of H_2_O_2_. The absorbance was measured at 517 nm on a Spectra Max® M5 Multi-mode Microplate Reader (Molecular Devices LLC.).

The ·OH scavenging ratio was calculate using the following formula:·OH scavenging effect (%) = (A_blank_− (A_extract_− A_control_)) /A_blank_× 100 where A_blank_ is the absorbance with distilled water instead of extract, A_extract_ is the absorbance with extract, and A_control_ is the absorbance with distilled water instead of H_2_O_2_.

### Amino acid analysis

#### Free amino acid analysis

Each sample (0.1 g) was placed in a 2-mL tube and dissolved in HCl (0.02 mol/L, 1 mL). After closing the tubes and mixing for 15 min at room temperature, the tubes were centrifuged at 12,000 × *g* for 15 min and the supernatant was collected. The supernatant (0.8mL) was mixed with 10% sulfosalicylic acid (0.8 mL) by vortexing for 2 min, and then after centrifuging at 12,000 × *g* for 15 min, the supernatant was collected. Subsequently, the supernatants were filtered through a 0.22-μm membrane for testing. Eventually, samples were analyzed by chromatography using a Hitachi L-8900 Automatic Amino Acid analyzer (Hitachi Ltd., Tokyo, Japan) fitted with a 2622SC-PF lithium citrate equilibrated ion exchange column (Hitachi Ltd.) and post column derivatization with ninhydrin, according to the manufacturer’s standard procedure [32].

#### Hydrolytic amino acid analysis

For hydrolytic amino acids, 0.1 g of each sample was placed in a 16 mm × 25 mm screw-cap tube and dissolved in 10 mL HCl (6 mol/L). The tubes were closed, placed in an electric oven at 110°C for 24 h, cooled, and then their content was diluted to 50 mL with ultrapure water. Then, 1mL of each diluted sample was removed and evaporated at 60°C until dry. The residue was then reconstituted with 2 mL of HCl (0.02 mol/L) and filtered through a 0.45-μm membrane. Subsequently, the contents of amino acids were analyzed using a Hitachi L-8900 Automatic Amino Acid analyzer (Hitachi Ltd.) fitted with a 2622SC-PH sodium citrate equilibrium ion exchange column (Hitachi Ltd.) and post column derivatization with ninhydrin, according to the manufacture’s standard procedure [32].

#### Flavor component analysis

The composition of flavor components was determined by the content of free amino acids [33, 34]. Total umami components (TUC) = aspartate + glutamate; total sweet components (TSC) = alanine + glycine + serine + threonine; total bitter components (TBC) = arginine + histidine + isoleucine + leucine + methionine + phenylalanine + valine; total tasteless components (TTC) = total amino acids (TAA) − TUC–TSC− TBC.

#### Essential amino acid composition

The amino acids ratio coefficient method [35, 36] was used to evaluate the essential amino acid compositions of FTDs and compared them with the essential amino acid model. The essential amino acid composition was calculated from the corresponding essential amino acids and TAA in the hydrolyzed amino acids.

### Statistical analysis

All analyses were conducted in SPSS 19.0. One-way ANOVAs followed by Duncan’s multiple comparison tests were used to assess differences between groups. Graphs were plotted with GraphPad Prism version 8.0 (GraphPad, USA). All parameter estimates were conducted in duplicate; the results were expressed as means and standard deviation. *P<*0.05 was considered significant.

## Results

### Characteristics of fermentation products

**Fig 1:**
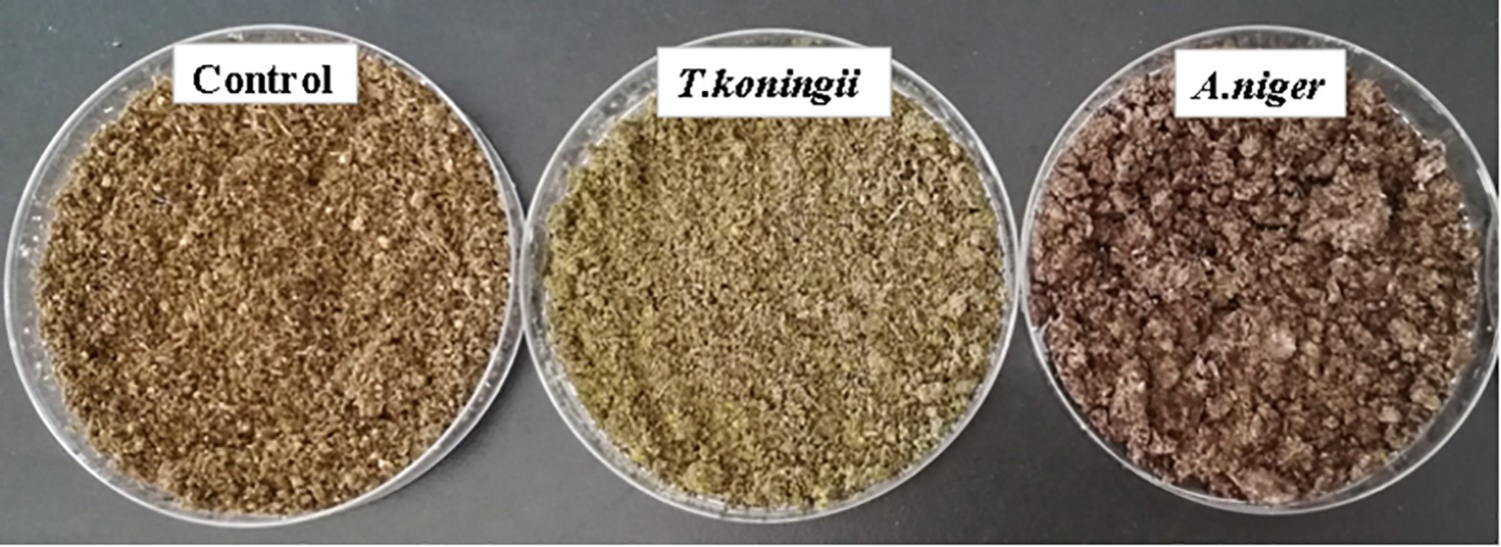
Characteristics (freeze-dried) in the fermented tea dregs.

The FTDs differed in color and structure (Fig 1). The TDs in the control, *T*. *koningii*, and *A*. *niger* were brownish yellow, green, and black, respectively. In the control and *T*. *koningii*, FTDs had a loose structure, while in *A*. *niger*, FTDs were large and had a tight structure. Compared with the control group, FTDs in *T*. *koningii* and *A*. *niger* were characterized by more pleasant and thicker tea fragrance (S2 Table).

### Proximate components and minerals

The proximate components and minerals of FTDs are shown in Table 1. The DM content was lower in *A*.*niger*, followed by *T*. *koningii* and the control with the highest (*P*< 0.05). The crude protein contents of TDs were 26.49 and 30.74% for the *T*. *koningii* and *A*. *niger* groups (*P*< 0.05), respectively, reflecting increases of 14.77 and 33.19%. The ether extract content of TDs was approximately 3% and was not affected by fungi (*P*> 0.05). Compared with the control, the contents of crude fiber, NDF, ADF, and ash in FTDs of *T*. *koningii* and *A*. *niger* were significantly increased (*P*< 0.05).The contents of crude fiber, NDF, and ADF in *T*. *koningii* FTDs were significantly higher than those in *A*. *niger* FTDs (*P*< 0.05). Compared with the control, the contents of carbohydrates in FTDs of the other two groups were significantly decreased (*P*< 0.05).

**Table 1:** Proximate components, minerals, and bioactive substances in the fermented tea dregs.

Items^1^	Control	*T*.*koningii*	*A*.*niger*	*P*-value
**Proximate components (g/kg)**				
**DM**	391 ± 3.5^a^	344 ± 6.3^b^	297 ± 6.87^c^	<0.001
**Crude protein**	230 ± 4.24^c^	265 ± 3.3^b^	307 ± 3.3^a^	<0.001
**Ether extract**	29 ± 4.0	33 ± 4.4	33 ± 4.0	0.242
**Crude fiber**	182 ± 3.4^c^	223 ± 3.8^a^	197 ± 12.2^b^	<0.001
**NDF**	439 ± 5.8^c^	493 ± 10.9^a^	463 ± 5.5^b^	<0.001
**ADF**	242 ± 6.5^c^	295 ± 5.1^a^	268 ± 22.6^b^	<0.001
**Ash**	32 ± 0.4^c^	38 ± 0.5^b^	41 ± 0.5^a^	<0.001
**Carbohydrates**	709 ± 2.4^a^	666 ± 6.8^b^	618 ± 4.1^c^	<0.001
**Minerals (mg/kg DM)**				
**Calcium**	5333 ± 68.4^b^	6378 ± 121.9^a^	6834 ± 90.2^a^	<0.001
**Phosphate**	6056 ± 68.4^b^	6699 ± 85.4^a^	6927 ± 88.0^a^	<0.001
**Potassium**	3969 ± 77.46^c^	4940 ± 54.6^b^	5332 ± 87.1^a^	<0.001
**Magnesium**	1610 ± 23.0^c^	2048 ± 80.6^b^	2192 ± 31.9^a^	<0.001
**Manganese**	970 ± 7.55^c^	1193 ± 16.0^b^	1311 ± 47.41^a^	<0.001
**Iron**	431 ± 14.8^c^	467 ± 7.9^b^	565 ± 12.6^a^	<0.001
**Zinc**	26 ± 1.2^c^	32 ± 0.8^b^	34 ± 1.2^a^	<0.001
**Copper**	10 ± 0.8^b^	14 ± 0.8^a^	15 ± 0.8^a^	<0.001
**Sodium**	ND	ND	ND	−
**Bioactive substances**				
**Reducing sugars (g/kg DM)**	7.40 ± 0.33^c^	17.96 ± 0.46^a^	14.43 ± 0.58^b^	<0.001
**Total polyphenols (gGAE/100 g DM)**	10.14 ± 0.33^b^	9.89 ± 0.36^b^	13.15 ± 0.67^a^	<0.001
**Total flavonoids (gRE/100 g DM)**	6.33 ± 0.22^b^	5.82 ± 0.40^b^	12.85 ± 0.49^a^	<0.001
**Theasaponins (mg/100 g DM)**	264.33 ± 16.40^b^	221.26 ± 3.35^c^	291.63 ± 17.52^a^	<0.001
**Caffeine (mg/100 g DM)**	124.16 ± 2.51^b^	146.22 ± 4.39^a^	ND	<0.001

Means with different letters in the same row (a–c) indicated a significant difference according to Duncan’s multiple comparison tests at *P*< 0.05.

^1^DM, dry mass; NDF, neutral detergent fiber; ADF, acid detergent fiber; ND, value under detection limits; GAE, gallic acid equivalent; RE, rutin equivalent.

In addition, compared with the control, the contents of calcium, phosphorus, potassium, magnesium, manganese, iron, zinc, and copper in FTDs of *T*. *koningii* and *A*. *niger* were significantly increased (*P*< 0.05). Also, the contents of potassium, magnesium, manganese, and iron in FTDs of *A*. *niger* was significantly higher than that of *T*. *koningii* (*P*< 0.05).

### Feeding value

As shown in Table 2, the feeding value of FTDs produced by *T*. *koningii* and *A*. *niger* was lower compared with the control (*P*< 0.05). The TDN, DDM, and RFV of FTDs were higher in our study than those in alfalfa [29], whereas DMI and RFQ were similar between alfalfa and FTDs.

**Table 2:** Feeding value of fermentation products.

Items^1^	Control	*T*. *koningii*	*A*. *niger*	*P*-value	Alfalfa^2^
**TDN (%DM)**	64.19 ± 0.48^a^	60.24 ± 0.38^c^	62.23 ± 0.27^b^	<0.001	56−58
**DDM(%DM)**	70.05 ± 0.50^a^	65.95 ± 0.40^c^	68.01 ± 0.28^b^	<0.001	47.23−49.25
**DMI (%DM)**	2.74 ± 0.04^a^	2.43 ± 0.06^c^	2.59 ± 0.03^b^	<0.001	2.88−3.19
**RFV**	149 ± 2.9^a^	125 ± 3.5^c^	137 ± 2.0^b^	<0.001	108−117
**RFQ**	143 ± 2.9^a^	119 ± 3.4^c^	131 ± 1.9^b^	<0.001	130−150

Means with different letters in the same row (a–c) indicated a significant difference according to Duncan’s multiple comparison tests at *P*< 0.05.

^1^ TDN, total digestible nutrient; DDM, digestibility of dry matter; DMI, dry matter intake; RFV, relative feed value; RFQ, relative forage quality.

^2^ Feeding value of alfalfa was according to Xiong et al. [29].

### Bioactive substances

The contents of bioactive substances were greatly affected by fungi in TDs (Table 1). The content of reducing sugar in FTDs of *T*. *koningii* and *A*. *niger* was higher than that in the control TDs by 142.7 and 95%, respectively (*P*< 0.05), and the content of reducing sugar was higher in *T*. *koningii* FTDs than in *A*. *niger* FTDs (*P*< 0.05). Additionally, the contents of total flavonoid and total polyphenol in *A*. *niger* FTDs were significantly higher than those in the *T*. *koningii* and control groups (*P*< 0.05). Compared with control TDs, the content of theasaponin in *T*. *koningii* FTDs was decreased (*P*< 0.05), while in *A*. *niger* FTDs it was increased (*P*< 0.05). Fermentation by fungi altered the content of caffeine in TDs. Caffeine content in TDs was completely degraded by *A*. *niger* (*P*< 0.05), whereas it was increased by 17.77% in *T*. *koningii* FTDs (*P*< 0.05) compared with the control.

### Antioxidant activity

The free radical scavenging ability of all samples was compared with that of vitamin C (Table 3). The DPPH free radical-scavenging ratio of the ethanol extract from FTDs of *A*. *niger* and *T*. *koningii* was significantly higher than that of the control (*P*< 0.05), but it was similar to that of vitamin C (*P*> 0.05). In addition, the •OH scavenging ratio of ethanol extract from *A*. *niger* FTDs was higher than that of the control and *T*. *koningii* (*P*< 0.05) but lower than that of vitamin C (*P*< 0.05). The highest scavenging ratio of ABST^+^ and •OH was that of vitamin C, followed by those of the methanol extracts of *A*. *niger*, *T*. *koningii*, and control TDs. The radical-scavenging ratio of vitamin C to ABST^+^ was higher than that of water extract from FTDs (*P*< 0.05), and the scavenging ratio of •OH in vitamin C and water extract from *A*. *niger* FTDs was higher than that of *T*. *koningii* (*P*< 0.05) and lowest in the control (*P*< 0.05).

**Table 3:** Free radical scavenging activity (%) of different solvent extracts from fermented tea dregs.

Items^1^	Vitamin C	Control	*T*.*koningii*	*A*.*niger*	*P*-value
**Ethanol extract**					
**ABTS** ^**+**^	90.36 ± 0.28^a^	86.72 ± 0.31^b^	86.84 ± 0.43^b^	87.48 ± 1.07^b^	<0.001
**DPPH**	88.19 ± 0.19^a^	64.17 ± 2.11^c^	82.47 ± 4.46^b^	80.89 ± 5.59^b^	<0.001
**•OH**	76.79 ± 2.11^a^	35.37 ± 3.57^c^	35.32 ± 2.70^c^	59.56 ± 4.89^b^	<0.001
**Methanol extract**					
**ABTS** ^**+**^	90.36 ± 0.28^a^	79.75 ± 0.82^d^	81.81 ± 1.43^c^	86.79 ± 0.87^b^	<0.001
**DPPH**	88.19 ± 0.19	85.03 ± 0.87	83.37 ± 2.41	84.21 ± 2.93	0.212
**•OH**	76.79 ± 2.11^a^	18.07 ± 2.13^d^	24.92 ± 1.42^c^	44.02 ± 1.51^b^	<0.001
**Water extract**					
**ABTS** ^**+**^	90.36 ± 0.28^a^	71.52 ± 0.57^b^	69.43 ± 1.17^c^	69.04 ± 1.82^c^	<0.001
**DPPH**	88.19 ± 0.19^a^	72.08 ± 7.08^b^	84.34 ± 8.19^a^	78.13 ± 9.95^ab^	0.045
**•OH**	76.79 ± 2.11^a^	20.06 ± 0.87^c^	28.54 ± 1.64^b^	76.00 ± 5.08^a^	<0.001

Means with different letters in the same row (a–c) indicated a significant difference according to Duncan’s multiple comparison tests at *P*< 0.05.

^1^ DPPH, 2, 2-diphenyl-1-picrylhy-drazyl; ABTS^**+**^, 2,2-azinobis-3-ethylben-zothiazoline-6-sulfonic acid diammonium salt.

### Amino acids

#### Free amino acids

**Fig 2:**
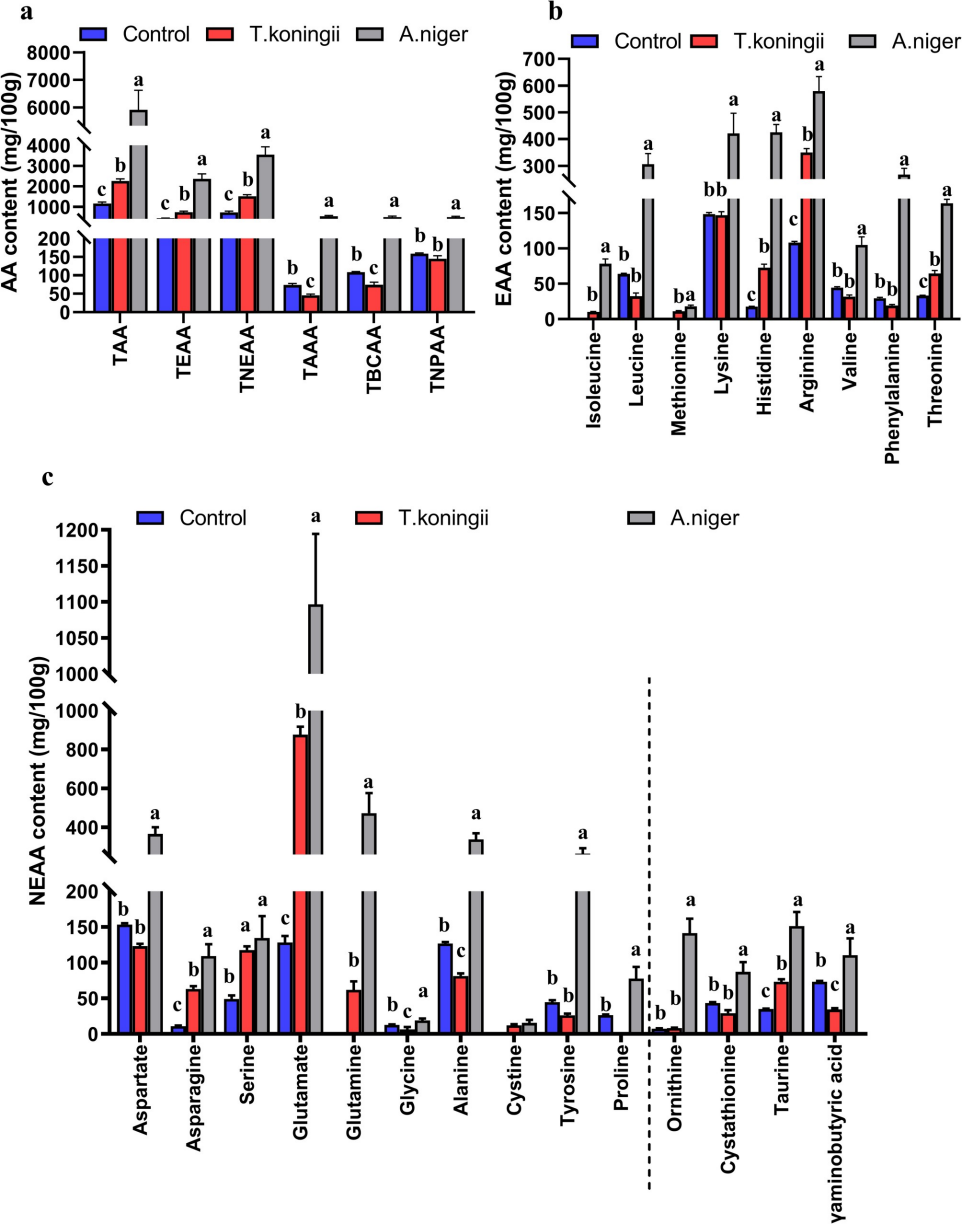
Free amino acids in the fermented tea dregs. TAA, total amino acids; TEAA, total essential amino acids; TNEAA, total non-essential amino acids; TAAA, total aromatic amino acids; TBCAA, total branched-chain amino acids; TNPAA, total non-protein amino acids. Values with the same letters were not significantly different (*P*< 0.05).

The content of free amino acids produced by TDs metabolized by fungi varied greatly (Fig 2). Specifically, there were significant differences in TAA, total essential amino acids, and total non-essential amino acids. *A*. *niger* had the highest content of TAA, followed by *T*. *koningii* and the control had the lowest (*P*< 0.05) (Fig 2A). The contents of total essential amino acids, total non-essential amino acids, total aromatic amino acids, total branched-chain amino acids, and total non-protein amino acids showed similar patterns of change to that of TAA in the treatments. The contents of all types of essential amino acids were the highest in *A*. *niger* FTDs (*P*< 0.05) (Fig 2B). The contents of histidine, arginine, and threonine were higher in the *T*. *koningii* FTDs than those in the control group (*P*< 0.05). The contents of non-essential amino acids were generally the highest in *A*. *niger* FTDs, while the contents of glycine were lower in *T*. *koningii*FTDs compared with the control group (*P*< 0.05). Compared with the control, *A*. *niger* increased the contents of non-protein amino acids (Fig 2C), while *T*. *koningii* decreased them. Moreover, taurine was improved by fermentation (*P*< 0.05).

#### Flavor components

The contents of umami and bitter amino acids in *T*. *koningii* and *A*. *niger* FTDs were higher than those in the control group (*P*< 0.05) (Table 4). Compared with the control and *T*. *koningii* groups, the contents of sweet and tasteless components in *A*. *niger* FTDs were higher (*P*< 0.05). The TUC/TAA ratio was higher in *T*. *koningii* FTDs than that in the other groups (*P*< 0.05), but the TTC/TAA ratio was the opposite. The TSC/TAA ratio in FTDs was lower than that in the unfermented control group (*P*< 0.05). The TBC/TAA ratio in *A*. *niger* FTDs was higher than those in the other groups (*P*< 0.05). Furthermore, the main flavor in *T*. *koningii* FTDs was umami, while in *A*. *niger* FTDs it was bitter.

**Table 4:** Flavored components in the fermented tea dregs.

Items^1^	Control	*T*.*koningii*	*A*.*niger*	*P*-value
**TUC (mg/100 g DM)**	281.67 ± 9.11^c^	999.06 ± 40.58^b^	1462.97 ± 177.63^a^	<0.001
**TSC (mg/100 g DM)**	222.25 ± 6.83^b^	270.31 ± 12.29^b^	655.77 ± 74.85^a^	<0.001
**TBC (mg/100 g DM)**	254.56 ± 11.87^c^	527.17 ± 27.75^b^	1780.04 ± 122.78^a^	<0.001
**TTC (mg/100 g DM)**	402.65 ± 59.89^b^	458.02 ± 60.82^b^	2017.03 ± 418.90^a^	<0.001
**TUC/TAA**	0.24 ± 0.02^b^	0.44 ± 0.02^a^	0.25 ± 0.02^b^	<0.001
**TSC/TAA**	0.19 ± 0.01^a^	0.12 ± 0.00^b^	0.11 ± 0.00^b^	<0.001
**TBC/TAA**	0.22 ± 0.01^b^	0.23 ± 0.01^b^	0.31 ± 0.02^a^	<0.001
**TTC/TAA**	0.35 ± 0.03^a^	0.20 ± 0.02^b^	0.34 ± 0.04^a^	<0.001

Means with different letters in the same row (a–c) indicated a significant difference according to Duncan’s multiple comparison tests at *P*< 0.05.

^1^ TAA, total amino acids; TUC, total umami components, aspartate + glutamate; TSC, total sweet components, alanine + glycine + serine + threonine; TBC, total bitter components, arginine + histidine + isoleucine + leucine + methionine + phenylalanine + valine; TTC, total tasteless components, TTC = TAA − TUC–TSC− TBC.

#### Hydrolyzed amino acids

**Fig 3:**
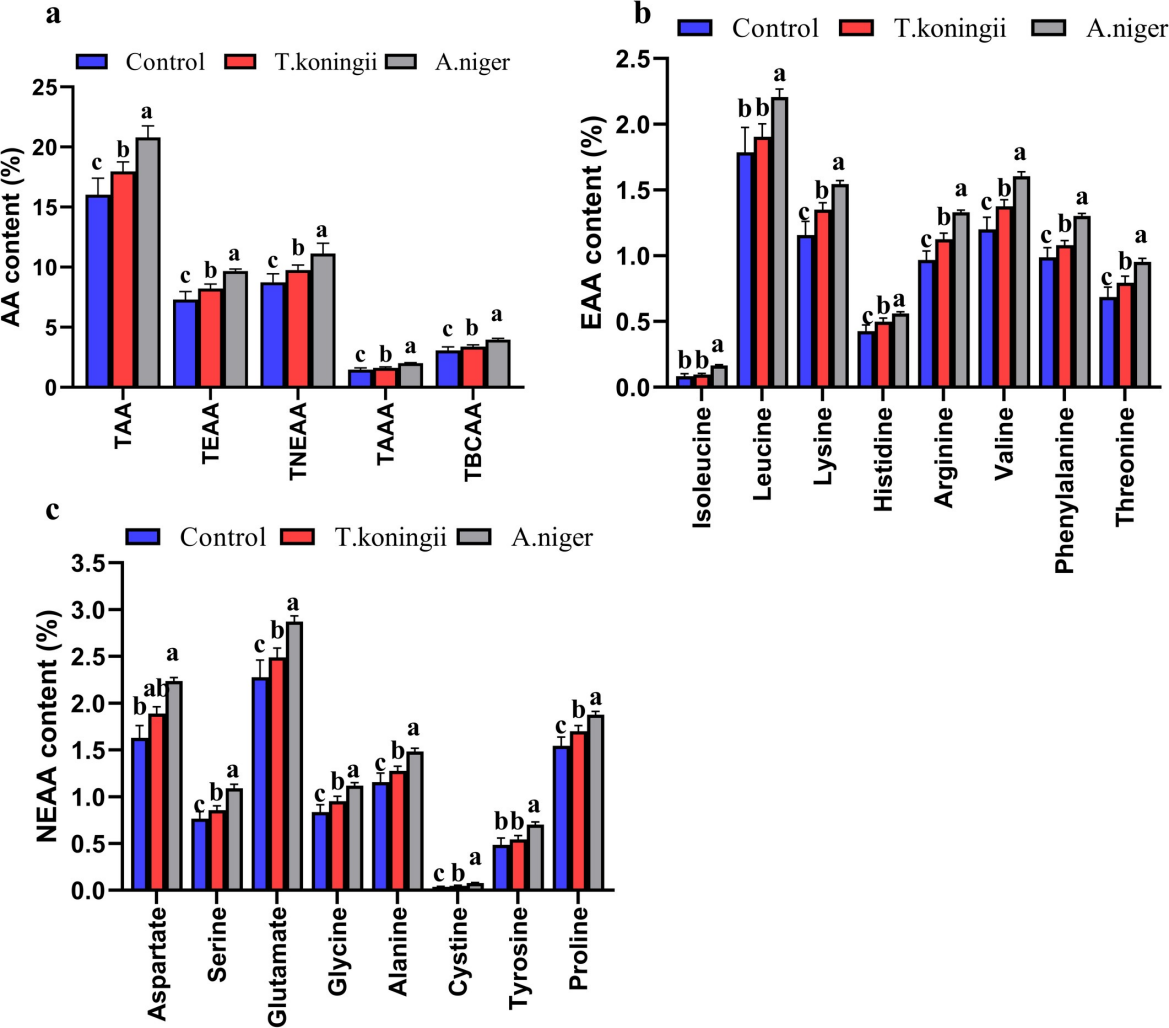
Hydrolytic amino acids in the fermented tea dregs. TAA, total amino acids; TEAA, total essential amino acids; TNEAA, total non-essential amino acids; TAAA, total aromatic amino acids; TBCAA, total branched-chain amino acids. Values with the same letters were not significantly different (*P*< 0.05).

The fungi caused great fluctuations in the content of hydrolyzed amino acids during fermentation (Fig 3). The highest levels of TAA, total essential amino acids, total non-essential amino acids, total aromatic amino acids, and total branched-chain amino acids were observed in *A*. *niger* FTDs, with levels that were significantly higher than those observed in *T*. *koningii* FTDs and the control groups (Fig 3A. As shown in Fig 3B, the highest contents of various essential amino acids (lsoleucine, leucine, lysine, histidine, arginine, valine, phenylalanine, threonine) were observed in *A*. *niger* FTDs (*P*< 0.05), followed by *T*. *koningii* FTDs and the control group had the lowest. Moreover, the contents of lysine, histidine, arginine, valine, phenylalanine, threonine in *T*. *koningii* FTDs were higher than those in the control TDs (*P*< 0.05). For non-essential amino acids (Fig 3C), the contents of serine, glutamate, glycine, alanine, cysteine, tyrosine, and proline were higher in *A*. *niger*and *T*. *koningii* FTDs than those in the control group(*P*< 0.05), and the contents of these amino acids (except tyrosine) were higher in *A*. *niger* FTDs than in *T*. *koningii* FTDs (*P*< 0.05).

#### Essential amino acid composition

As shown in Table 5, the composition of threonine and tyrosine + phenylalanine were higher in *A*. *niger* FTDs than those in *T*. *koningii*and control groups (*P*< 0.05). The composition of lysine was higher in *A*. *niger* FTDs and *T*. *koningi i*than in the control group (*P*< 0.05). Improved effects of fungal fermentation on the composition of all essential amino acids except for cystine + methionine and isoleucine. In addition, the proportion of total essential amino acids in different fermentation products was 45.7−46.3%, which was slightly lower than that in egg protein, but higher than the Food and Agriculture Organization of the United Nations mode [36].

**Table 5:** Essential amino acids composition of fermentation products.

Items^1^	Control	*T*.*koningii*	*A*.*niger*	*P*-value	Egg protein^2^	FAO pattern^3^
**Lysine**	7.2 ± 0.05^b^	7.5 ± 0.04^a^	7.4 ± 0.29^ab^	0.029	6.4	5.5
**Cystine +Methionine**	0.2 ± 0.02^c^	0.3 ± 0.02^b^	0.4 ± 0.03^a^	<0.001	5.5	3.5
**Threonine**	4.3 ± 0.12^b^	4.4 ± 0.09^ab^	4.6 ± 0.18^a^	0.005	5.1	4
**Valine**	7.5 ± 0.07	7.7 ± 0.05	7.7 ± 0.31	0.123	7.3	5
**Leucine**	11.2 ± 0.24^a^	10.6 ± 0.10^b^	10.6 ± 0.40^b^	0.006	8.8	7
**Isoleucine**	0.5 ± 0.08^b^	0.5 ± 0.03^b^	0.8 ± 0.02^a^	<0.001	6.6	4
**Tyrosine+Phenylalanine**	9.2 ± 0.12^b^	9.0 ± 0.09^b^	9.6 ± 0.36^a^	0.001	10	6
**TEAA**	45.5 ± 0.29	45.8 ± 0.11	46.5 ± 1.85	0.275	49.7	35

Means with different letters in the same row (a–c) indicated a significant difference according to Duncan’s multiple comparison tests at *P*< 0.05.

^1^ TEAA, total essential amino acids.

^2^ Egg protein was according to Li et al. [35].

^3^ FAO pattern, the Food and Agriculture Organization of the United Nations, was according to FAO/WHO [36].

## Discussion

The color of the FTDs were consistent with the colors of fungal spores. FTDs structural differences may stem from the development of *A*. *niger* hyphae and also that the fermented product had a relatively tight structure. A loose structure is beneficial for the dispersion of TDs in the feed. After several soaking and extraction cycles, the TDs lost most of its fragrance. This study found that fermentation with *A*. *niger* and *T*. *koningii* improved the aroma of tea dregs, which indicated that fermentation can transform some components of tea into volatile compounds, some of which are considered important contributors to the aroma of tea [9, 37]. The increase in aroma may help increase the attractiveness of TDs to animals and improve the palatability of TDs. It also increases the possibility of FTDs as human tea beverages.

There were considerable differences in the chemical composition of *T*. *koningii* and *A*.*niger* FTDs. Microorganisms produce water by aerobic respiration. Thus, high water content may indicate that *A*. *niger* would be more suitable for fermenting TDs than *T*. *koningii*. SSF by *T*. *koningii* and *A*. *niger* affected the crude protein content by facilitating the production of bacterial proteins, suggesting that these TDs could be used as an alternative source of animal feed. In fact, SSF has been previously shown to be an efficient and environmentally-friendly method for producing by-products or waste by facilitating the production of high amounts of crude protein, such as in ginkgo leaves [30] and rapeseed cake [38]. The ether extract value in our study was higher than that of a previous study reporting the ether extract content of green TDs (1%) [9]. Ultimately, estimates of ether extract content depend on the processing technology used as well as the source and type of TDs. In this study, the content of fiber components were increased by fermentation. The NDF and ADF contents of the control group were similar to those in green TDs [9]. The fungal cell wall is an important structure outside the fungal cell, and is primarily composed of dextran, mannan, and chitin [39]. Chitin, one of the most important components of the cell wall, was found to show the most variation in content between strains [40]. Fungal cell wall components (β-Glucan, chitin, and chitosan) are classified as dietary fibers that promote animal health [40]. When fungi grow in large quantities, chitin content also increases, which may lead to increase in fiber content. As dietary fiber is beneficial to animal health, the increase in dietary fiber in FTDs may enhance their viability as feed additives and facilitate their widespread use in the future. The decrease of carbohydrates indicates that the fungi use carbohydrates in FTDs as energy source to generate amino acids, synthesize proteins, nucleic acids, and fats [41]. There is a corresponding relationship between the carbohydrate content and crude protein content [42]. As the crude protein content is increased by fungi, the corresponding carbohydrate content, is decreased.

The increase of the ash content in FTDs was consistent with the increase of the minerals content. Fungal hyphae are abundant and there are various kinds of metabolic enzymes, which can synthesize phytase, which may degrade phytate phosphorus and phytate mineral complex in TDs, resulting in the increase of the mineral content [43]. Moreover, the mineral content increasing effect of *A*. *niger* is better than that of *T*. *koningii*. Another suggestion is that during the fermentation process, carbohydrates are decomposed to produce carbon dioxide, alcohol and volatile organic acids, which reduces the content of substrates, leading to a relative increase in the crude protein, ether extract, ash, and mineral content. This is consistent with the reduction of the DM of FTDs.

The relative feed value is one of the most widely used indices for predicting the quality of feed for ruminants [44]. The TDN, DDM, and RFV of FTDs were higher in our study than in those reported for alfalfa [29], and DMI and RFQ were similar between alfalfa and the FTDs in our study. As expected, both TDs and FTDs were suitable ruminant feed.

Reducing sugarsare the final product of many biological processes and enzymatic reactions and primarily consist of glucose, fructose, and galactose [45, 46]. Measuring the concentration of reducing sugars in the samples can provide valuable information, such as the amount of sugar in the different foods and the activity of enzymes that degrade cellulose and starch. Cellulase produced by fungal metabolism can catalyze the conversion of cellulose into reducing sugars, such as monosaccharides. In this study, *T*. *koningii* and *A*. *niger* fermentation increased the reducing sugar content. *T*. *koningii* FTDs had higher content of reducing sugars than *A*. *niger* FTDs, which suggests that the enzyme activities of *T*. *koningii* were higher than those in *A*. *niger*. Monosaccharides are a type of soluble sugar that can partially suppress the bitter taste of TDs [47]. The taste of the *T*. *Koningii* FTDs may thus be superior to that of *A*. *niger* FTDs. In addition, the increased content of reducing sugars in TDs is beneficial to the digestion, absorption, and energy supply of animals.

Flavonoid and polyphenol compounds have strong antioxidant capacity, which is one of the main properties facilitating improvements in the intestinal and immune function of animals [48, 49]. The contents of total flavonoids and total polyphenols in *A*. *niger* FTDs were higher than those in the *T*. *koningii* and the control groups, which is consistent with the findings of Wang et al. [30] and Dulf et al. [50], who found that *A*. *niger* increased the total flavonoid content of ginkgo leaves and apricot press residues by 26 and more than 30%, respectively. *A*. *niger* is a valuable microorganism for the biotransformation of flavonoids and polyphenols leading to the enhancement of the antioxidant activity [51, 52]. Moreover, total flavonoids and total polyphenols are the most important components contributing to the bitterness and astringency of tea [10]. *A*. *niger* FTDs with high total flavonoid and total polyphenol contents indicated that the residues fermented by *A*. *niger* tasted more bitter.

Saponins usually exhibit antioxidant, immunomodulatory, and antifungal activities [11, 53]. However, high levels of saponins can reduce the absorption of nutrients in animals, thus limiting the use of saponins in feed [54].The metabolism of microorganisms is considered to be the most promising treatment of degradation of anti-nutritional factors. In this study, the content of theasaponins was reduced by *T*. *koningii*. Few studies have been conducted on *T*. *koningii*, and no literature to date has suggested that *T*. *koningii* can degrade saponins. Ren et al. [55] found that *A*. *niger* had a positive effect on the degradation of theasaponins, which is inconsistent with our study showing that *A*. *niger* increased the content of theasaponins. This inconsistency may stem from the different fermentation substrates (TDs vs. camellia oil meal) that were used in these studies.

Caffeine is the most abundant alkaloid in tea, and contributes to its bitterness and astringency [10], and is an environmental pollutant [56]. Caffeine was found to be degraded by *A*. *niger* in our study, which is inconsistent with the findings of Zhou et al. [57], who confirmed the ability of *A*. *niger* fermentation to increase the caffeine content. Another study found that the caffeine degradation ability of *A*. *niger* was limited in the presence of glucose and other nutrients [58]. Caffeine degradation ability may related to *A*.*niger* strain, fermentation substrate, culture conditions and so on. The decrease in the caffeine content increases the palatability of TDs for animals and also reduces potential damage to the environment. However, moderate amounts of caffeine have been shown to have positive impacts on health [59], as it has antioxidant, anti-diabetic, and anti-obesity activities [11]. Also, as a central nervous system stimulant, the effects of caffeine are well established [10]. In contrast to *A*. *niger*, the caffeine content was increased by *T*. *koningii*. FTDs of *T*. *koningii* could thus be used as feed material for animals in moderation, as these FTDs could excite the central nervous system of animals and enhance blood circulation.

Measurement of DPPH, ABTS and OH radicals is widely used to evaluate the antioxidant capacity of biological samples [60]. In this study, the antioxidant capacity of FTDs was slightly higher than that of unfermented, which may be related to the concentration of the extract from TDs or FTDs. To a certain extent, fermentation by *A*. *niger* improved the antioxidant capacity of TDs. The antioxidant activity differed among different solvent extracts, likely due to the difference in the contents of various active substances among the different solvents. The antioxidant activity of different extracts of FTDs is also closely related to the content of flavonoids, polyphenols, theasaponins, and caffeine. Therefore, the combination of multiple bioactive substances affects the antioxidant activity of FTDs. Feeding FTDs with certain antioxidant capacity can promote animal health and improve growth performance.

Free amino acids are important bioactive components of tea [61]. The content of TAA in the TDs was in the range of 1655−2499 mg/100 g [62], and the free amino acids in the TDs increased rapidly after fermentation. Leucine, methionine, cysteine, and glutamine were produced by fungi, especially *A*. *niger*. Indeed, the contents of various free amino acids were the most abundant in *A*. *niger*, which may be due to the rich enzyme system of *A*. *niger* [63], its high protease activity, and cellulose content. The protein and cellulose in TDs are decomposed by fungi to produce a variety of amino acids. In fact, the peptides and proteins in tea can be used by microorganisms during fermentation and result in further changes in the abundances of certain free amino acids [64]. Additionally, taurine was improved by fermentation. Thus, the metabolic pathways of amino acids of *A*. *niger* and *T*. *koningii* to use TDs differed. Noteworthy, *L*-theanine was not detected in TDs and could not be produced by fermentation. During fermentation, proteins may decompose, polymerize, or convert, resulting in major changes in amino acids content and composition. Free amino acids are good sources of nutrients, such as vitamin E, folic acid, and other nutrients, for animals. These free amino acids can be directly absorbed and used, reflecting the nutritional value of food. The nutritional value of TDs was improved by fermentation with either *A*. *niger* or *T*. *koningii*.

Flavored amino acids can make foods have sweet, bitter, and umami tastes and contribute to a rich taste hierarchy of food [34]. Grouping the amino acids with taste characteristics [33] revealed that the content of various flavors amino acids was highest in *A*. *niger* FTDs. Notably, the main flavor amino acids in *T*. *koningii* FTDs was umami, and in *A*. *niger* FTDs was bitter, indicating that the flavor of FTDs differed between these fungi. Combined with the results of reducing sugars, total flavonoids, and total polyphenols, the taste of *A*. *niger* FTDs was more bitter than that of *T*. *koningii* FTDs. Therefore, it is necessary to consider the effect of palatability on feed intake and pay attention to the dosage of FTDs when used as supplement for feeding animals.

The contents of different species of amino acids in TDs increased to varying degree after fermentation, which is consistent with the findings of previous studies [65]. Different amino acids often showed similar pattern of increasing content after fermentation. The abundant content of amino acids in *A*. *niger* and *T*. *koningii*FTDs was likely the result of its high crude protein content. The increase in the crude protein was primarily attributed to the growth of fungal mycelium. The estimated levels of crude protein and hydrolyzed amino acids were consistent. The fermentation effect of *A*. *niger* on TDs was thus more beneficial than that of *T*. *koningii*on TDs. The quality of essential amino acids is a core indicator of nutritional value. Animals are limited to the biosynthesis of certain amino acids (i.e., non-essential amino acids), while the remaining (essential) amino acids must be obtained through food. The composition of lysine, threonine, valine, leucine, tyrosine and phenylalanine were better or close to those of the standard model [36]. The results of this study showed that the protein quality of FTDs was good and an excellent source of protein feed. However, the proportions of sulfur-containing amino acids and isoleucine were much lower than those observed in these two models. Thus, sulfur-containing amino acids and isoleucine might need to be supplemented when FTDs are used as feed materials.

## Conclusion

Solid-state fermentation with *Aspergillus niger* and *Trichoderma koningii* effectively improved the nutritional value of tea dregs, increased the content of functional substances, and improved the flavor of tea dregs. *A*. *niger* formed fermented tea dregs with especially beneficial effects. Fermented tea dregs contain active ingredients and have environmentally friendly properties. Thus, fermented tea dregs could be used as a low-cost, high-value animal feed additive and could even be incorporated into human diets. Further animal and toxicity tests are required to determine the safety of fermented tea dregs as animal feed.

## Supporting information

S1 File Mycotoxin content in fermentation products. (PDF)

S1 Table Criteria for odor quality assessment. (DOCX)

S2 Table Odor scores from fermented tea dregs. (DOCX)

S1 Dataset Experiment dataset. (XLSX)
