# Supplementary material for: Solid-state fermentation by Aspergillus niger and Trichoderma koningii improves the quality of tea dregs for use as feed additives
Source: PLoS One. 2021 Nov 12;16(11):e0260045. doi: 10.1371/journal.pone.0260045 (PMC8589212; doi:10.1371/journal.pone.0260045)
Supplement: S1 File — (PDF) [file pone.0260045.s001.pdf]

Quantitative Analysis Sample Report

**Batch Data Path**	H:\MassHunter\Data\2021\08\AF\20210824\QuantResults\20210824.batch.bin		
**Analysis Time**	8/25/2021 2:10 PM	**Analyst Name**	KMKZ-EA03-02\Admin
**Report Time**	8/25/2021 2:27 PM	**Reporter Name**	KMKZ-EA03-02\Admin
**Last Calib Update**	8/25/2021 2:10 PM	**Batch State**	Processed

AFB1

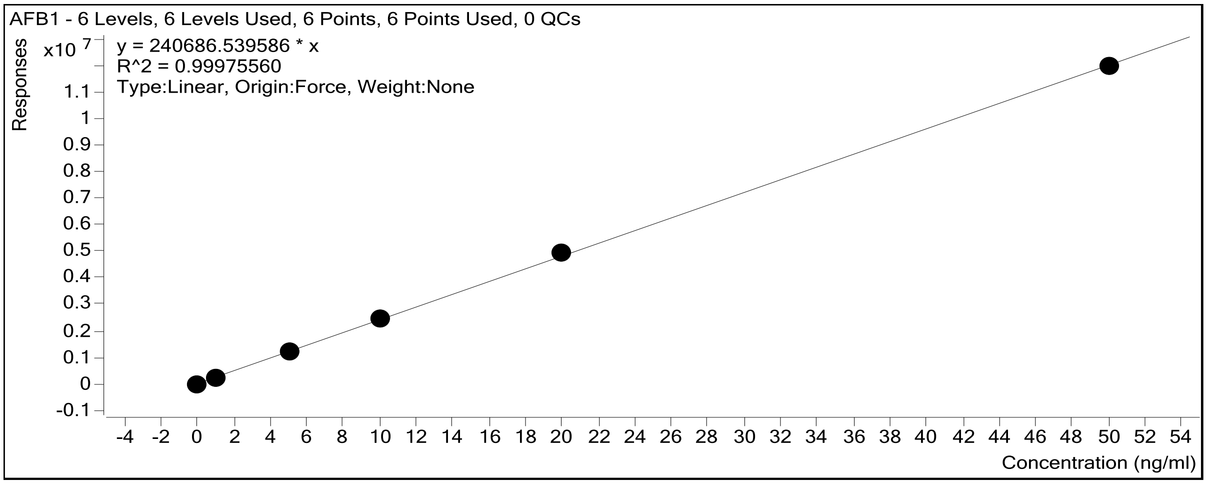

Data	Acq Time	Type	Name	Compound	ISTD	RT	Final Conc		Resp	ISTD Resp
003.d	2021/8/24 11:43	Sample	SJKB	AFB1		3.977	0.0000	ng/ml	0	
004.d	2021/8/24 11:50	Sample	GCKB	AFB1		3.977	0.0000	ng/ml	0	
007.d	2021/8/24 12:10	Calibration	STD-1	AFB1		3.955	0.0252	ng/ml	6059	
008.d	2021/8/24 12:16	Calibration	STD-2	AFB1		3.955	0.8574	ng/ml	206362	
009.d	2021/8/24 12:23	Calibration	STD-3	AFB1		3.955	4.9468	ng/ml	1190618	
010.d	2021/8/24 12:30	Calibration	STD-4	AFB1		3.955	10.3227	ng/ml	2484529	
011.d	2021/8/24 12:37	Calibration	STD-5	AFB1		3.955	20.4938	ng/ml	4932589	
012.d	2021/8/24 12:43	Calibration	STD-6	AFB1		3.955	49.7461	ng/ml	11973218	
014.d	2021/8/24 12:57	Sample	WS2104719006-ZK	AFB1		3.955	5.0413	ng/ml	1213373	
028.d	2021/8/24 16:32	Sample	WS2104719006 -TDs	AFB1		4.325	0.0000	ng/ml	0	
029.d	2021/8/24 16:39	Sample	WS2104719007-T. koningii FTDs	AFB1		4.336	0.0000	ng/ml	0	
030.d	2021/8/24 16:46	Sample	WS2104719008-A.niger FTDs	AFB1		4.056	0.0000	ng/ml	0	
032.d	2021/8/24 17:01	Sample	STD-3-RR	AFB1		3.955	4.2267	ng/ml	1017320	

SJKB

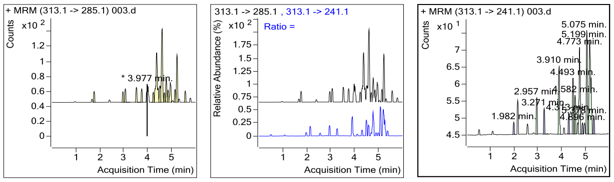

GCKB

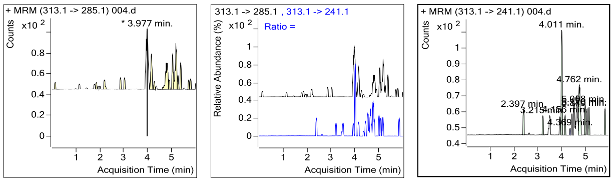

STD-1

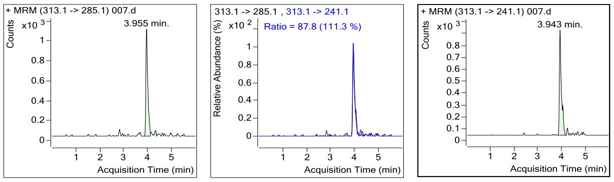

STD-2

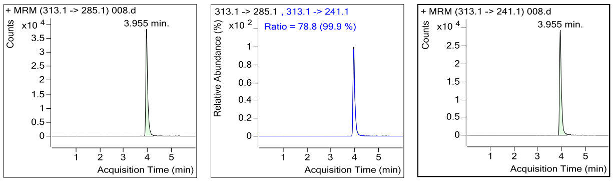

Trier: Checked by: Review date: .

STD-3

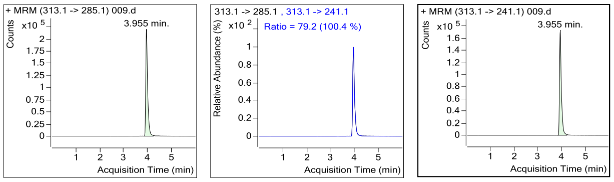

STD-4

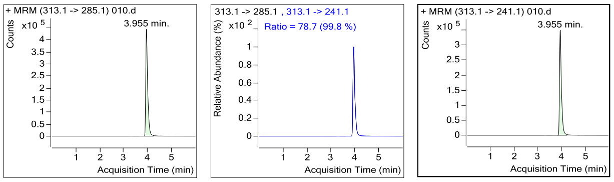

STD-5

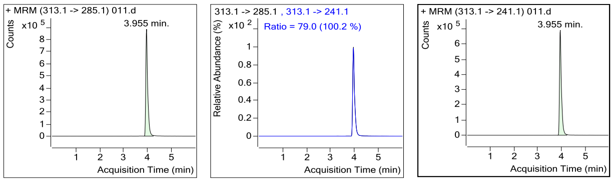

STD-6

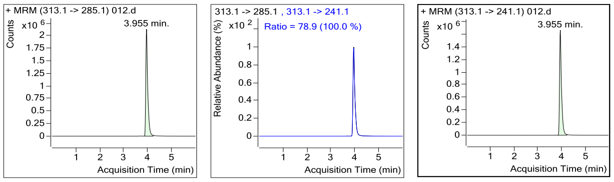

WS2104719006-ZK

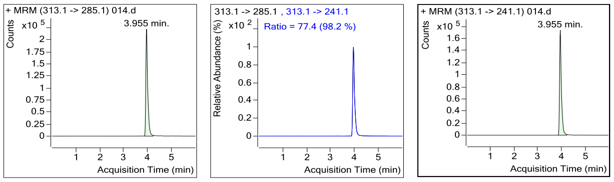

WS2104719006

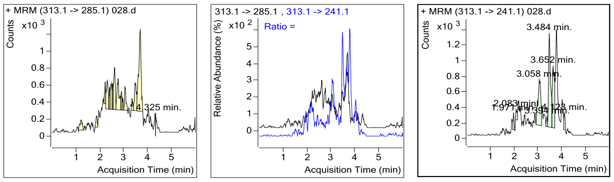

WS2104719007

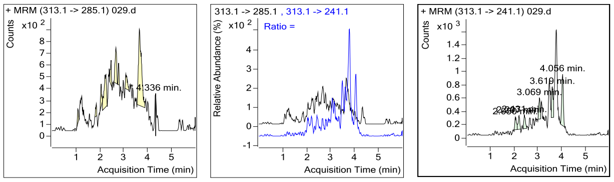

WS2104719008

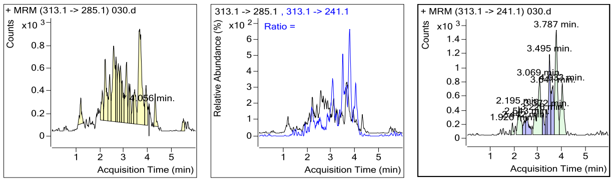

STD-3-RR

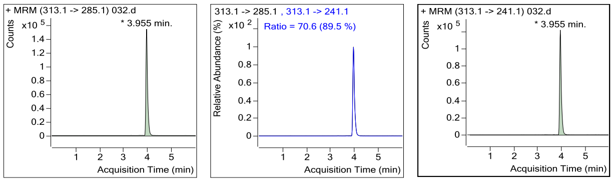

Trier: Checked by: Review date: .

T-2 toxin

Batch Name: XT-20210901.dab

	Sample Name	Sample Type	Acquisition Date	Analyte Peak Name	Analyte Peak Area (counts)	Analyte Peak Height (cps)	Analyte Concentration (ng/mL)	Calculated Concentration (ng/mL)	Accuracy (%)
1	std-10ppb	Standard	9/4/2021 4:07:22 PM	T-2	2.38e+004	3.91e+003	10.0	11.5	115.
2	std-20ppb	Standard	9/4/2021 4:12:46 PM	T-2	4.59e+004	7.09e+003	20.0	22.2	111.
3	std-30ppb	Standard	9/4/2021 4:18:14 PM	T-2	7.02e+004	1.25e+004	30.0	34.0	113.
4	std-40ppb	Standard	9/4/2021 4:23:40 PM	T-2	8.78e+004	1.50e+004	40.0	42.4	106.
5	std-50ppb	Standard	9/4/2021 4:29:07 PM	T-2	9.20e+004	1.43e+004	50.0	44.5	89.0
6	add-20ppb	Quality Control	9/4/2021 4:34:31 PM	T-2	4.38e+004	7.17e+003	20.0	21.2	106.
7	kb	Unknown	9/4/2021 4:39:59 PM	T-2	0.00e+000	0.00e+000	N/A	No Peak	N/A
8	2108032831-1 -TDs	Unknown	9/4/2021 4:45:27 PM	T-2	0.00e+000	0.00e+000	N/A	No Peak	N/A
9	2108032831-2	Unknown	9/4/2021 4:50:53 PM	T-2	0.00e+000	0.00e+000	N/A	No Peak	N/A
10	2108032832-1 -T. koningii	Unknown	9/4/2021 4:56:21 PM	T-2	0.00e+000	0.00e+000	N/A	No Peak	N/A
11	2108032832-2 FTDs	Unknown	9/4/2021 5:01:48 PM	T-2	0.00e+000	0.00e+000	N/A	No Peak	N/A
12	2108032833-1	Unknown	9/4/2021 5:07:13 PM	T-2	0.00e+000	0.00e+000	N/A	No Peak	N/A
13	2108032833-2 -A.niger FTDs	Unknown	9/4/2021 5:12:42 PM	T-2	0.00e+000	0.00e+000	N/A	No Peak	N/A

郑实柔　　　　刘福友

Batch Name: XT-20210901.dab

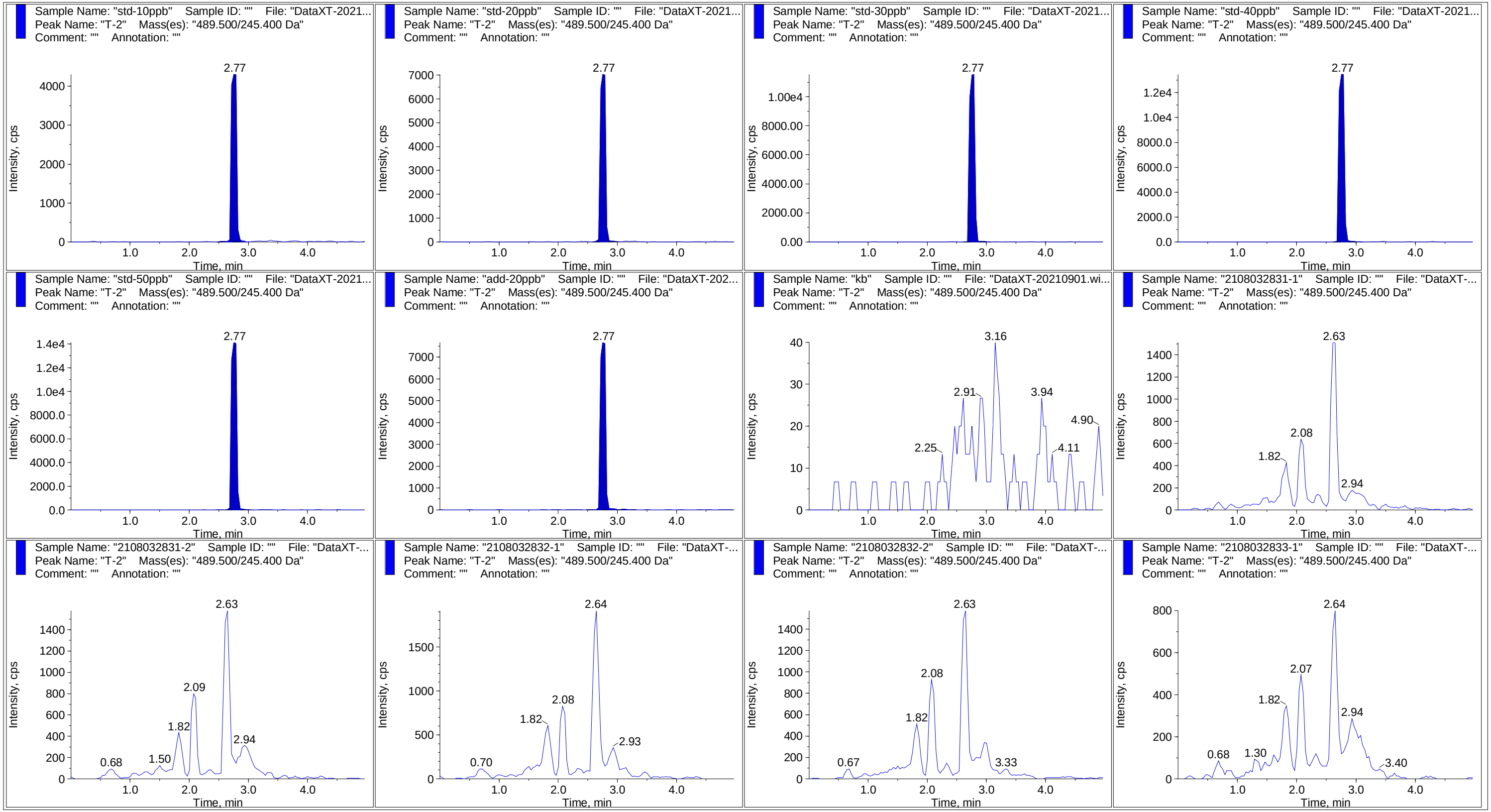

郑奕柔 胡福友

Sample Name: "2108032833-2" Sample ID: "" File: "DataXT-...
Peak Name: "T-2" Mass(es): "489.500/245.400 Da"
Comment: "" Annotation: ""

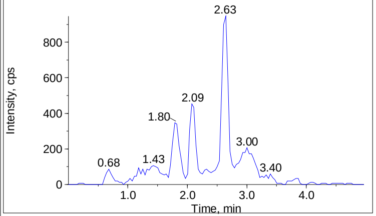

郑英柔　　刘福友

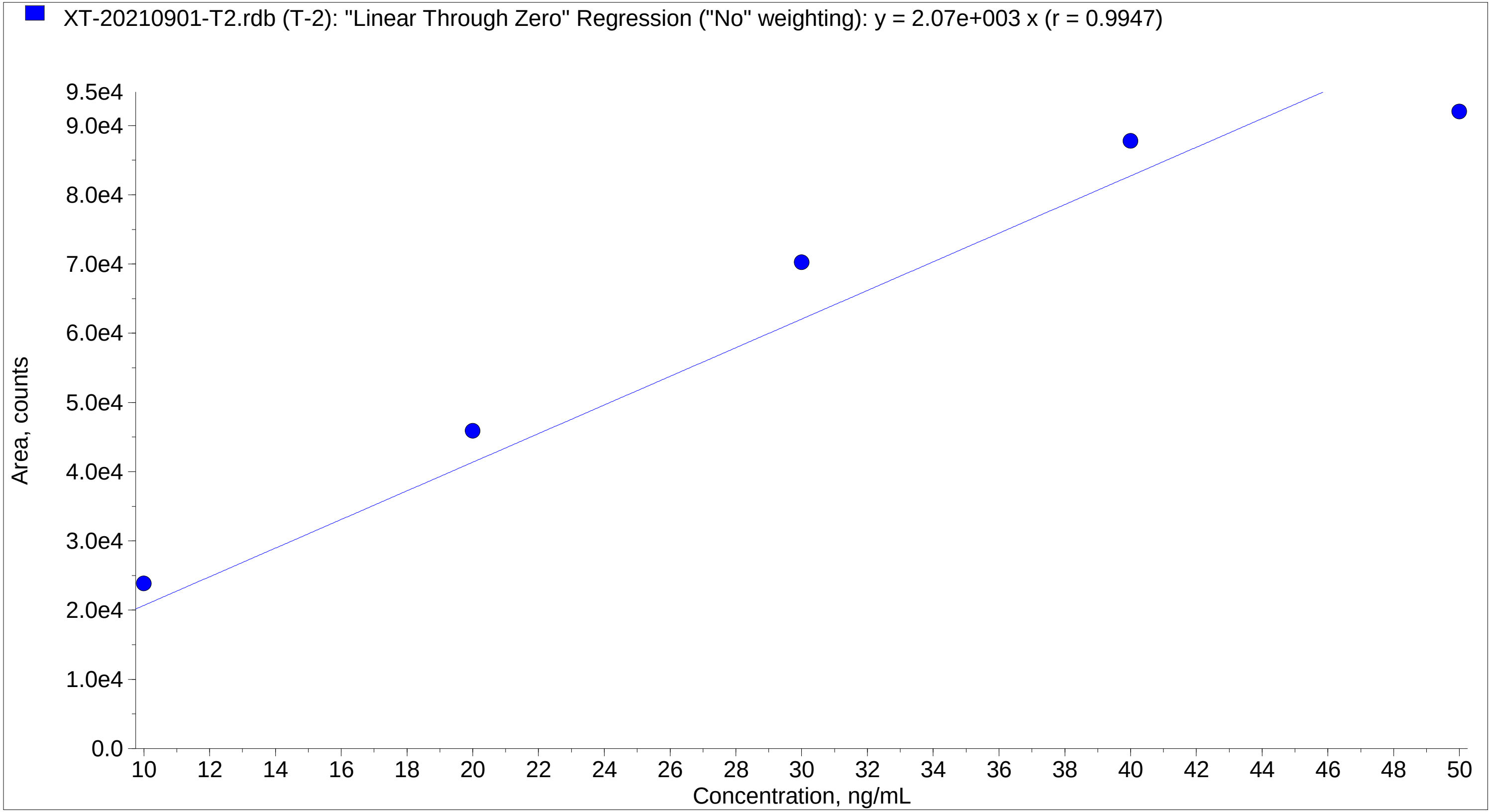
XT-20210901-T2.rdb (T-2): "Linear Through Zero" Regression ("No" weighting): y = 2.07e+003 x (r = 0.9947)
9.5e4
9.0e4
8.0e4
7.0e4
6.0e4
5.0e4
4.0e4
3.0e4
2.0e4
1.0e4
0.0
Area, counts
10
12
14
16
18
20
22
24
26
28
30
32
34
36
38
40
42
44
46
48
50
Concentration, ng/mL

郑实柔

刘福友

**Batch Data Path**	H:\MassHunter\Data\2021\08\ZEN\20210824\QuantResults\20210824.batch.bin		
**Analysis Time**	8/25/2021 2:32 PM	**Analyst Name**	KMKZ-EA03-02\Admin
**Report Time**	8/25/2021 2:33 PM	**Reporter Name**	KMKZ-EA03-02\Admin
**Last Calib Update**	8/25/2021 2:32 PM	**Batch State**	Processed

ZEN

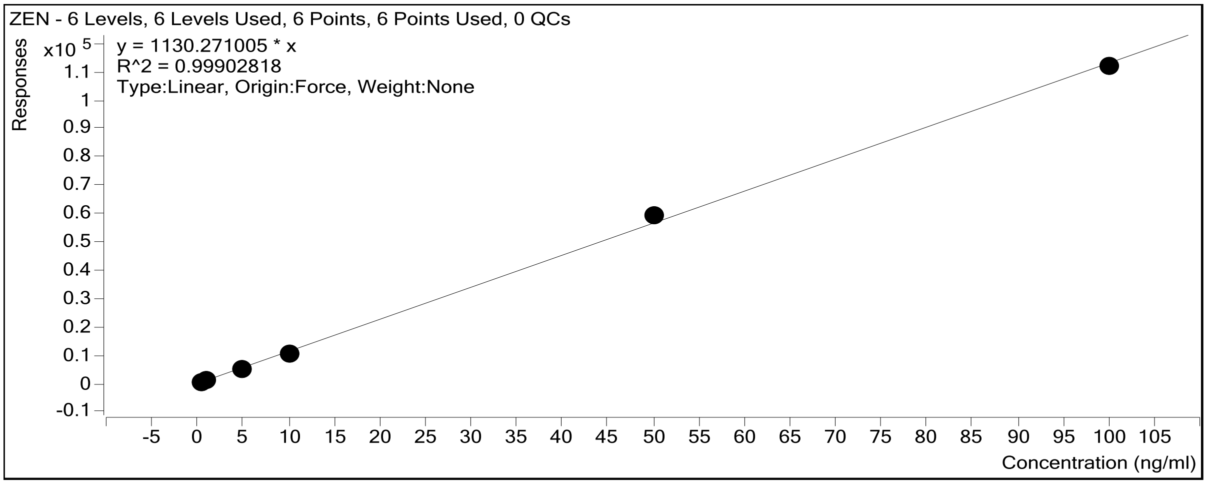

Data	Acq Time	Type	Name	Compound	ISTD	RT	Final Conc		Resp	ISTD Resp
002.d	2021/8/24 17:17	Sample	SJKB	ZEN		3.017	0.0000	ng/ml	0	
003.d	2021/8/24 17:22	Sample	GCKB	ZEN		3.226	0.0000	ng/ml	0	
005.d	2021/8/24 17:34	Calibration	STD-1	ZEN		3.151	0.3363	ng/ml	380	
006.d	2021/8/24 17:40	Calibration	STD-2	ZEN		3.151	0.8812	ng/ml	996	
007.d	2021/8/24 17:45	Calibration	STD-3	ZEN		3.151	4.5018	ng/ml	5088	
008.d	2021/8/24 17:51	Calibration	STD-4	ZEN		3.151	9.5068	ng/ml	10745	
009.d	2021/8/24 17:57	Calibration	STD-5	ZEN		3.151	52.4388	ng/ml	59270	
010.d	2021/8/24 18:02	Calibration	STD-6	ZEN		3.151	98.8569	ng/ml	111735	
012.d	2021/8/24 18:14	Sample	WS2104719006-ZK	ZEN		3.151	58.6614	ng/ml	66303	
013.d	2021/8/24 18:20	Sample	WS2104719006 -TDs	ZEN		3.143	0.0000	ng/ml	0	
014.d	2021/8/24 18:25	Sample	WS2104719007-T. koningii FTDs	ZEN		3.151	0.0000	ng/ml	0	
015.d	2021/8/24 18:31	Sample	WS2104719008-A.niger FTDs	ZEN		3.117	0.0000	ng/ml	0	
023.d	2021/8/24 19:24	Sample	STD-4-R	ZEN		3.159	9.9469	ng/ml	11243	

SJKB GCKB

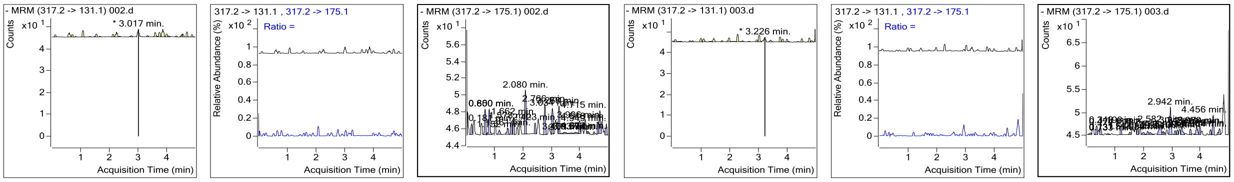

STD-1 STD-2

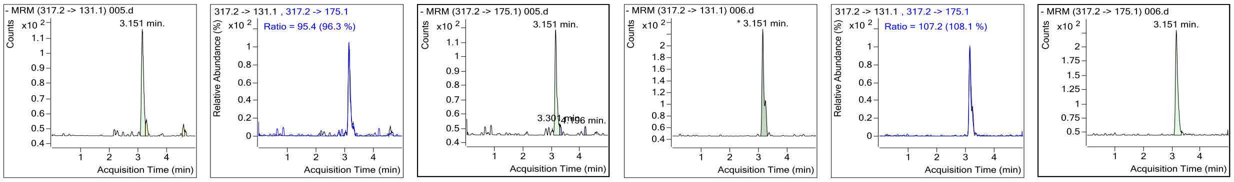

Trier: Checked by: Review date: .

STD-3

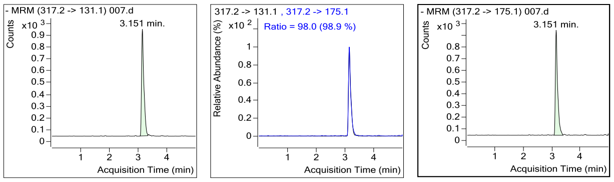

STD-4

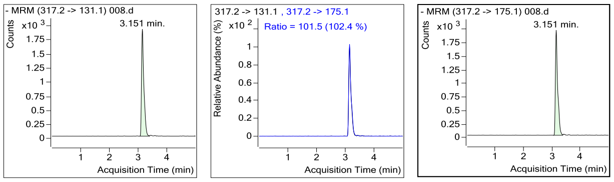

STD-5

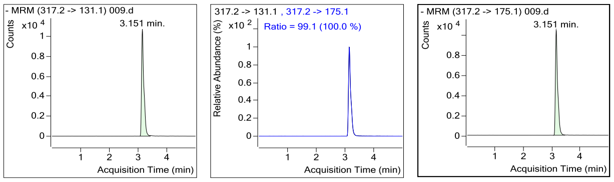

STD-6

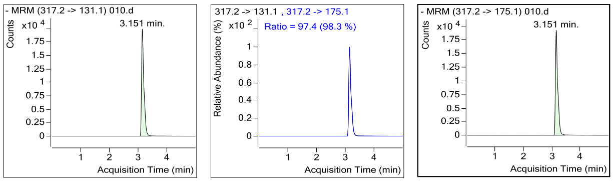

WS2104719006-ZK

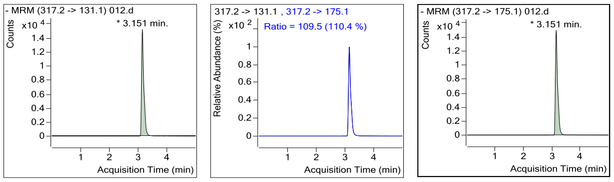

WS2104719006

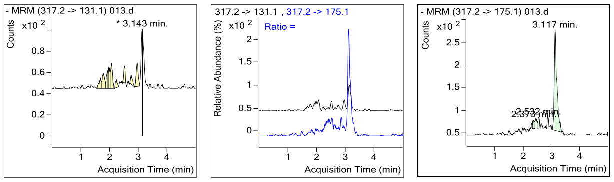

WS2104719007

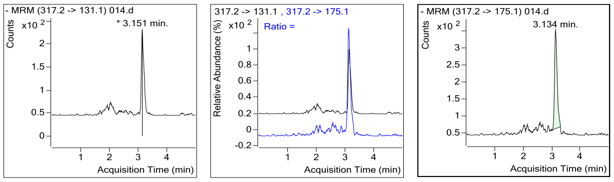

WS2104719008

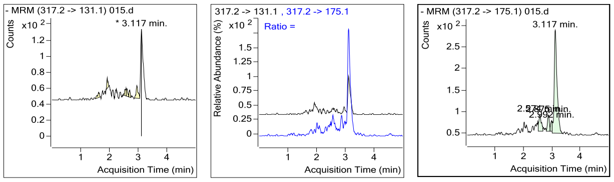

STD-4-R

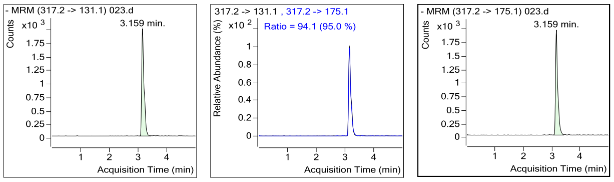

Trier: Checked by: Review date: .

Deoxynivalenol

样品统计报告

序列 采集者	test	序列 采集日期	9/1/2021 9:33:26 AM
采集方法	呕吐毒素.amx	数据 分析方法	*呕吐毒素.pmx
采集方法修改日期	6/20/2021 11:16:20 AM	仪器 名称	KMKZEA01-01

化合物: 呕吐毒素

信号: DAD1A

预期保留时间: 5.924

相关系数.: 0.999681

残留: 1.73705

RF RSD%:

公式: y = ax + b

a: 78.81751

b: 0.44612

c: 0.00000

d:

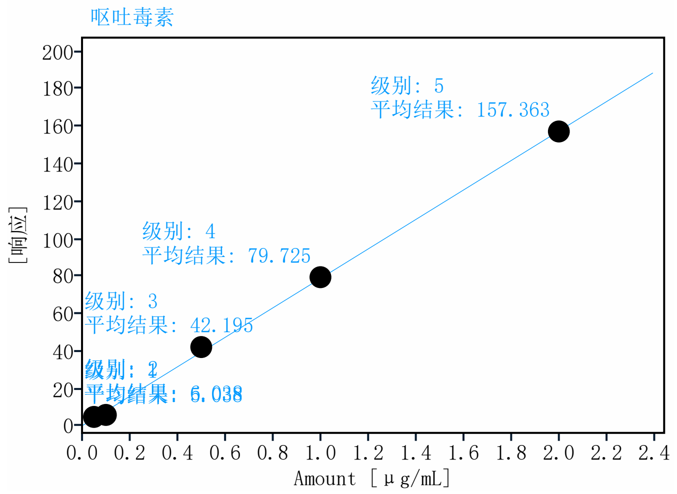

样品：SJKB；信号：DAD1A,Sig=218.0,4.0 Ref=360.0,100.0

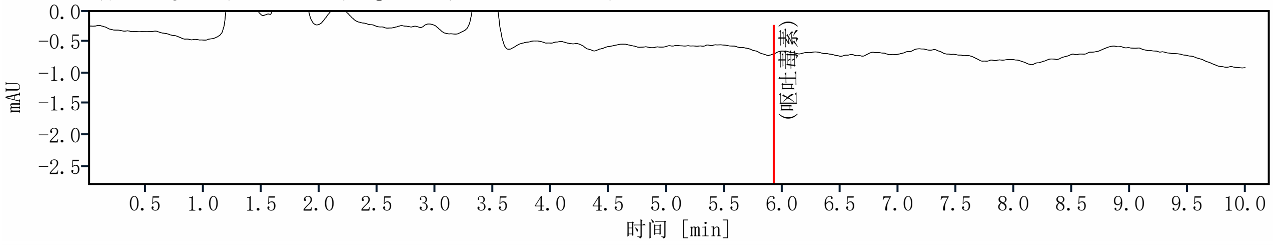

样品：GCKB；信号：DAD1A,Sig=218.0,4.0 Ref=360.0,100.0

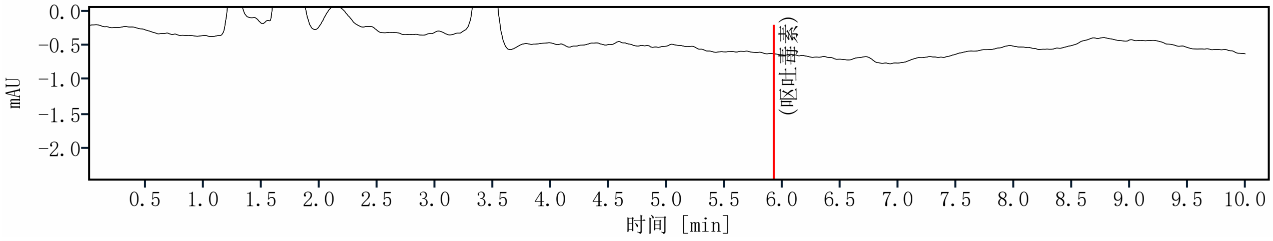

样品：STD-1；信号：DAD1A,Sig=218.0,4.0 Ref=360.0,100.0

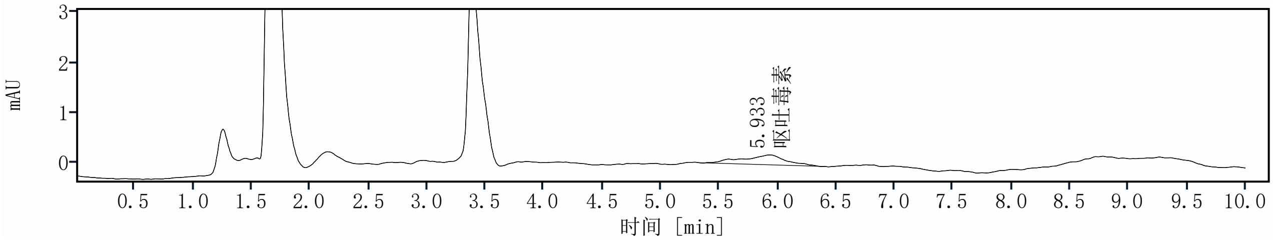

检验员: 复核人: 复核日期:

样品统计报告

样品：STD-2；信号：DAD1A,Sig=218.0,4.0 Ref=360.0,100.0

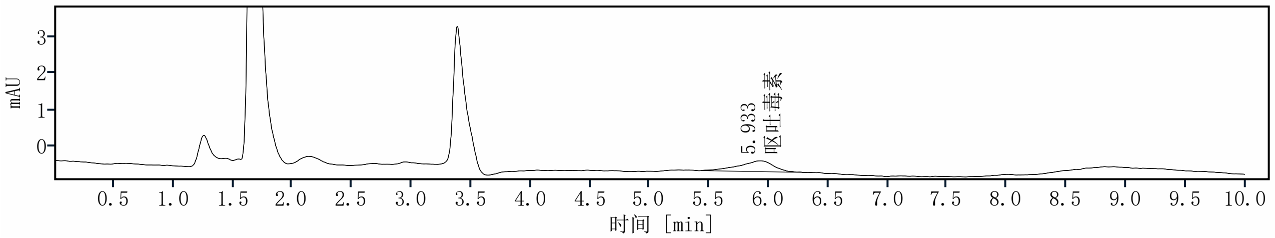

样品：STD-3；信号：DAD1A,Sig=218.0,4.0 Ref=360.0,100.0

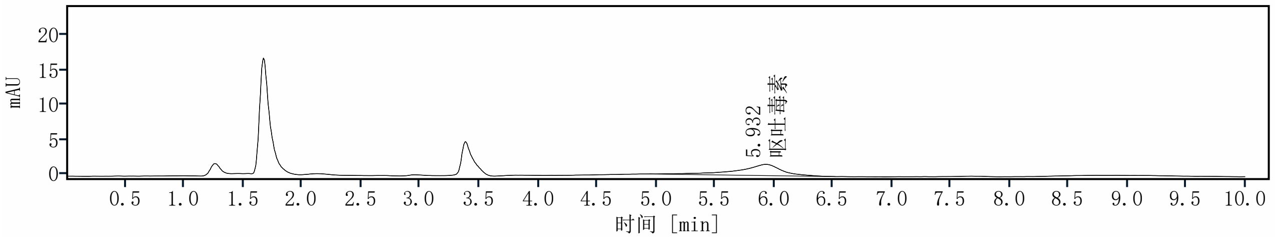

样品：STD-4；信号：DAD1A,Sig=218.0,4.0 Ref=360.0,100.0

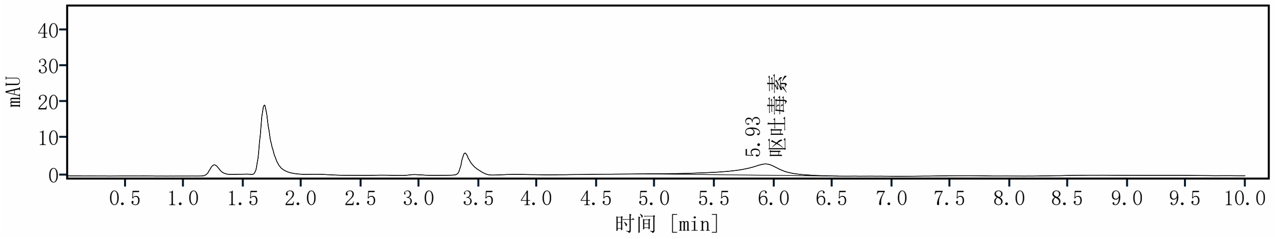

样品：STD-5；信号：DAD1A,Sig=218.0,4.0 Ref=360.0,100.0

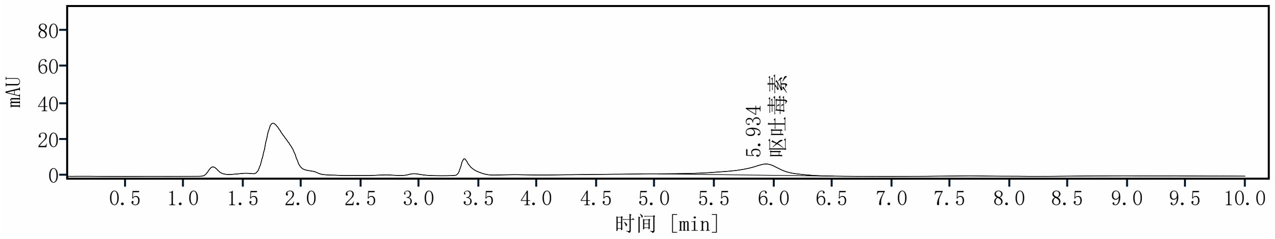

样品：STD-6；信号：DAD1A,Sig=218.0,4.0 Ref=360.0,100.0

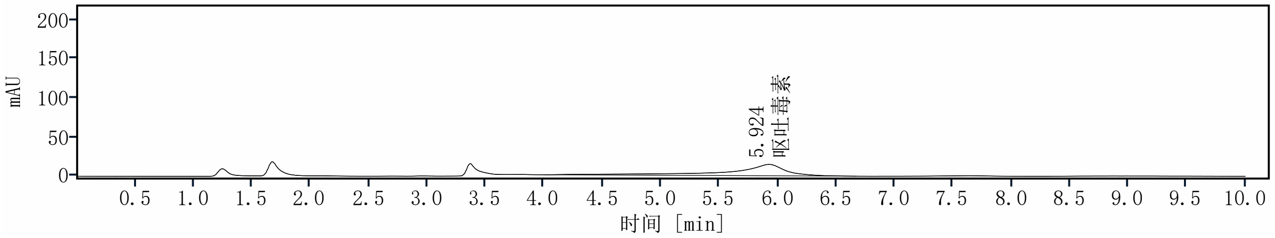

样品：WS2104896001；信号：DAD1A,Sig=218.0,4.0 Ref=360.0,100.0

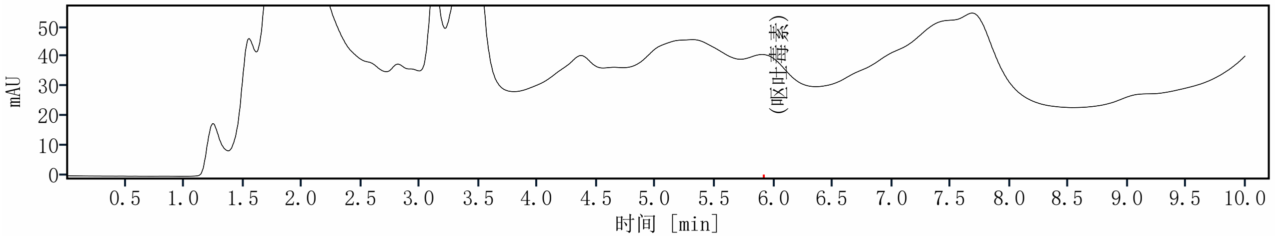

检验员:　　复核人:　　复核日期:

样品统计报告

样品：WS2104896002；信号：DAD1A,Sig=218.0,4.0 Ref=360.0,100.0

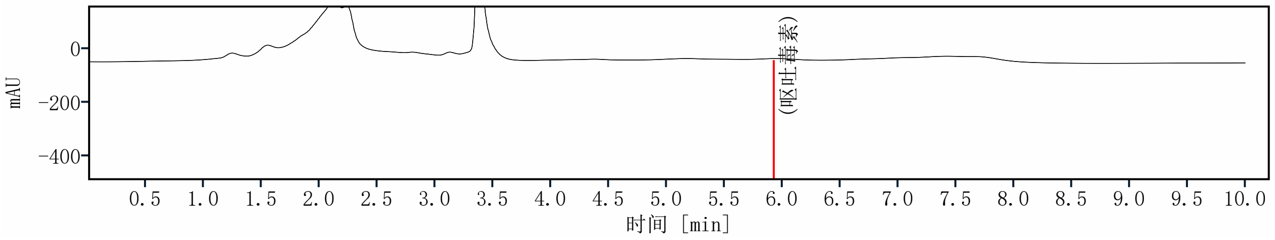

样品：WS2104896003；信号：DAD1A,Sig=218.0,4.0 Ref=360.0,100.0

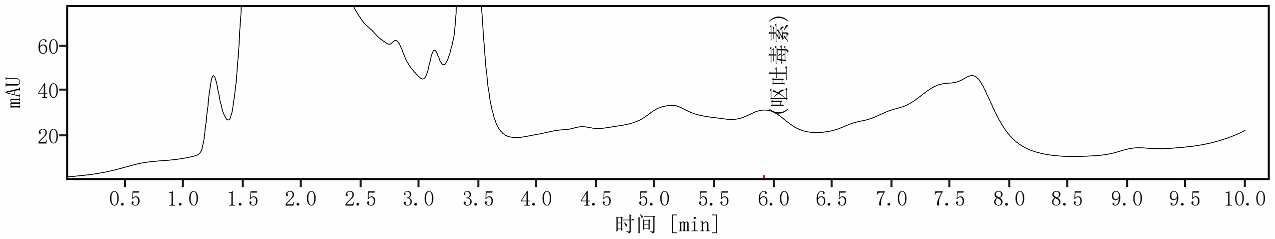

样品：STD-4-R；信号：DAD1A,Sig=218.0,4.0 Ref=360.0,100.0

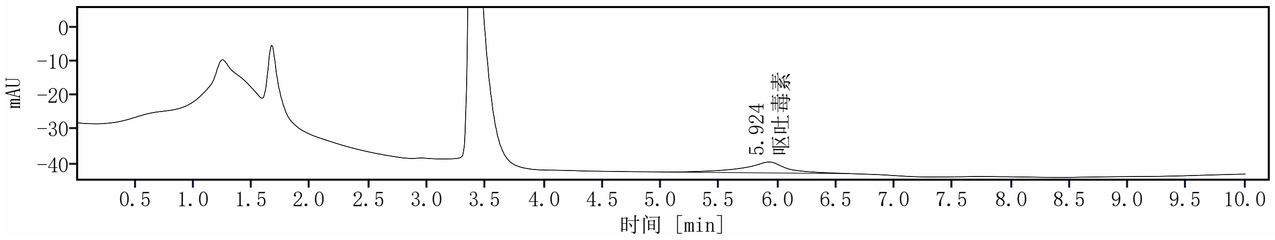

名称 呕吐毒素

样品名称	样品瓶号	保留时间[min]	面积	峰高[pA]	采集日期	含量[μg/mL]
SJKB	31	5.924	0.000	0.000	09/01/2021 09:35:15	0.0000
GCKB	32	5.924	0.000	0.000	09/01/2021 09:47:05	0.0000
STD-1	33	5.933	5.038	0.198	09/01/2021 09:58:49	0.0583
STD-2	34	5.933	6.038	0.299	09/01/2021 10:10:35	0.0710
STD-3	35	5.932	42.195	1.620	09/01/2021 10:22:22	0.5297
STD-4	36	5.930	79.725	3.098	09/01/2021 10:34:14	1.0059
STD-5	37	5.934	157.363	6.228	09/01/2021 10:45:57	1.9909
STD-6	38	5.924	491.203	14.487	09/01/2021 10:57:42	6.2265
WS2104896001 -TDs	39	5.924	0.000	0.000	09/01/2021 11:21:14	0.0000
WS2104896002 -T. koningii FTDs	40	5.924	0.000	0.000	09/01/2021 11:33:02	0.0000
WS2104896003 -A.niger FTDs	41	5.924	0.000	0.000	09/01/2021 11:44:48	0.0000
STD-4-R	36	5.924	83.286	3.187	09/01/2021 12:31:48	1.0510

检验员: 复核人: 复核日期:

Ochratoxin

样品信息

分析者 : System Administrator
设备编号 : GZ20210203
样品架号 : 1
样品瓶号 : 1
进样体积 : 20 uL
数据文件名 : 试剂空白.lcd
方法文件名 : 20210902.lcm
批处理文件名 : 20210902-1.lcb
分析日期 : 2021/9/2 12:44:33

汇总(每个化合物)
试剂空白.lcd

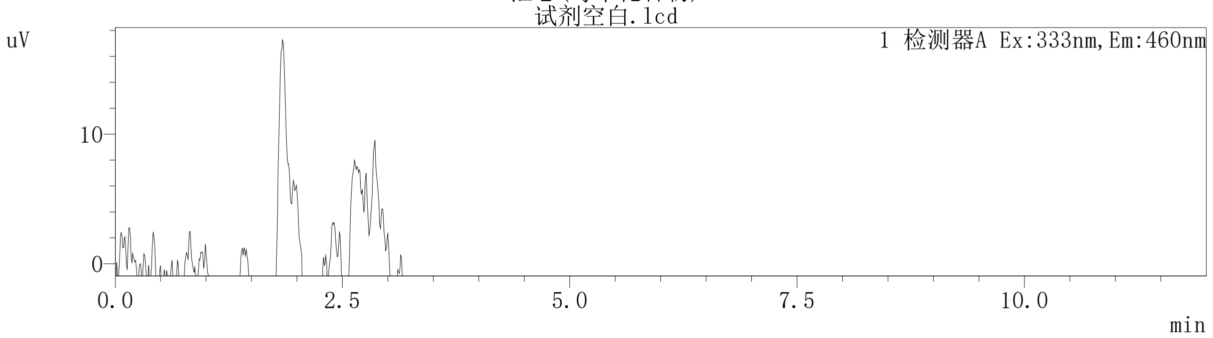

STD-1.lcd

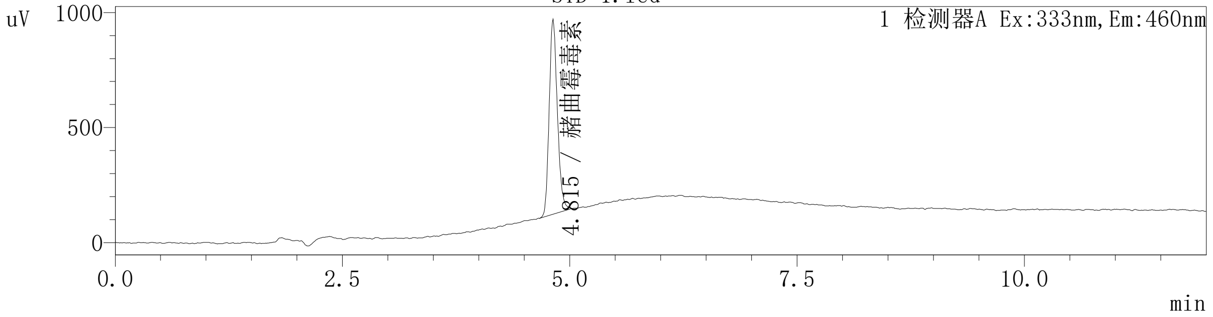

STD-2.lcd

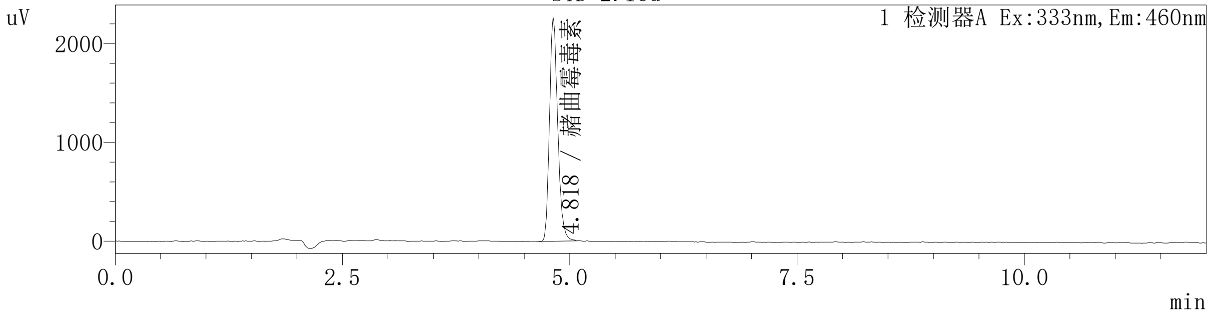

STD-3.lcd

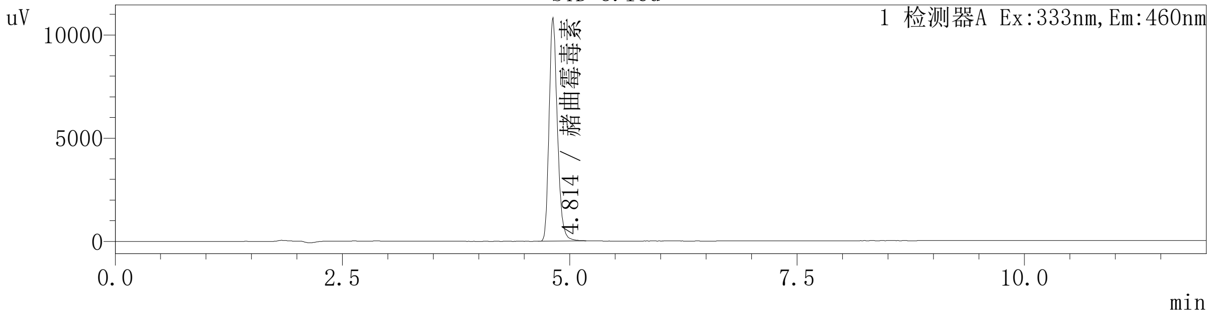

 检验者：　　复核者：　　复核日期：

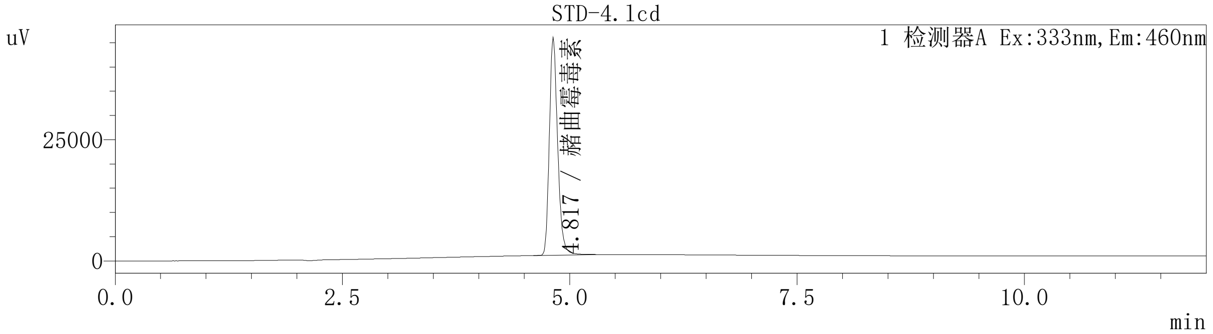
STD-4.lcd
uV
1 检测器A Ex:333nm,Em:460nm
4.817 / 赭曲霉毒素
25000
0
0.0
2.5
5.0
7.5
10.0
min

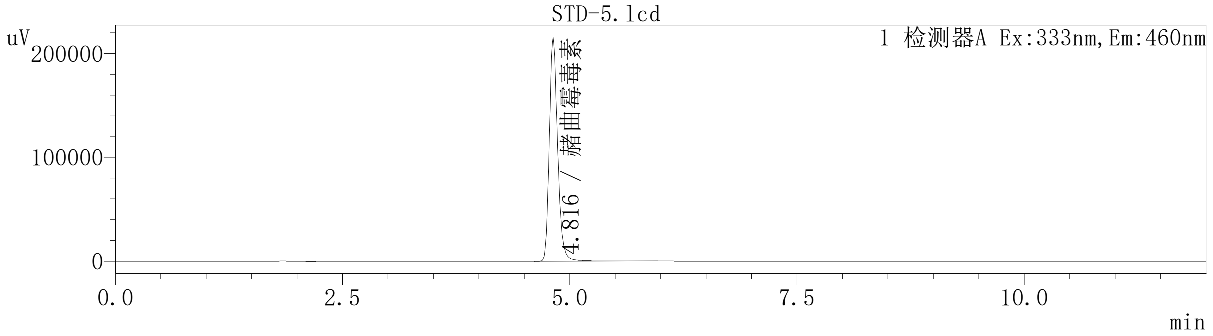
STD-5.lcd
uV
1 检测器A Ex:333nm,Em:460nm
4.816 / 赭曲霉毒素
200000
100000
0
0.0
2.5
5.0
7.5
10.0
min

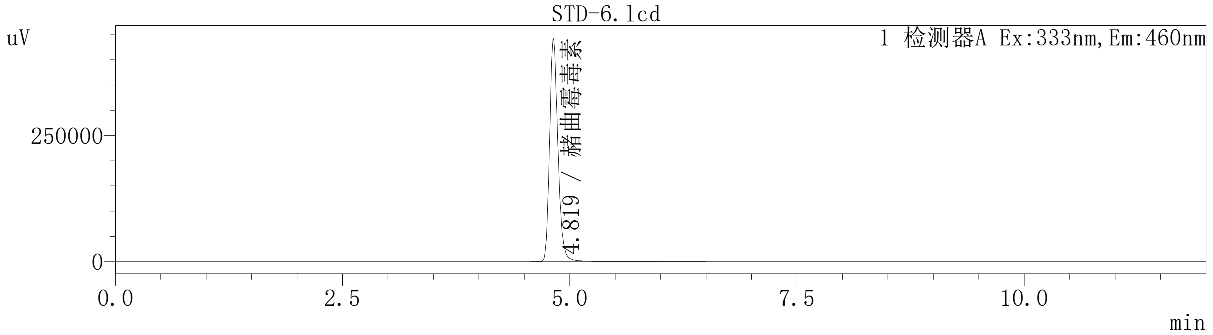
STD-6.lcd
uV
1 检测器A Ex:333nm,Em:460nm
4.819 / 赭曲霉毒素
250000
0
0.0
2.5
5.0
7.5
10.0
min

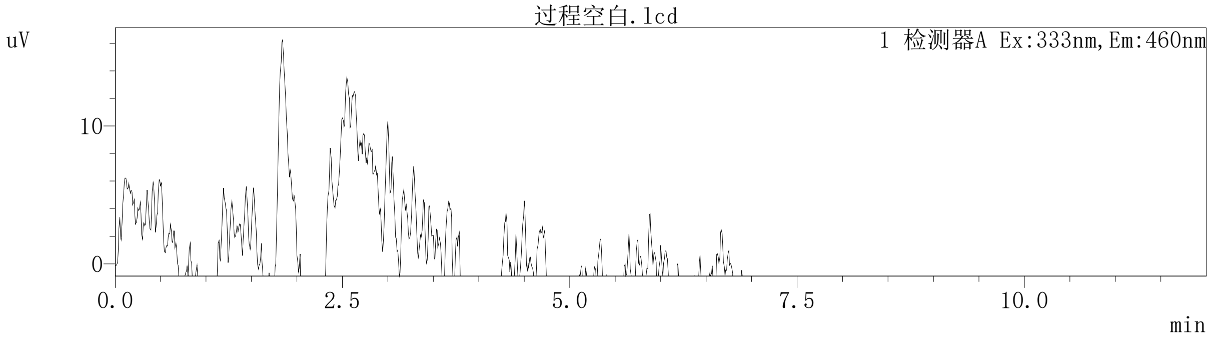
过程空白.lcd
uV
1 检测器A Ex:333nm,Em:460nm
10
0
0.0
2.5
5.0
7.5
10.0
min

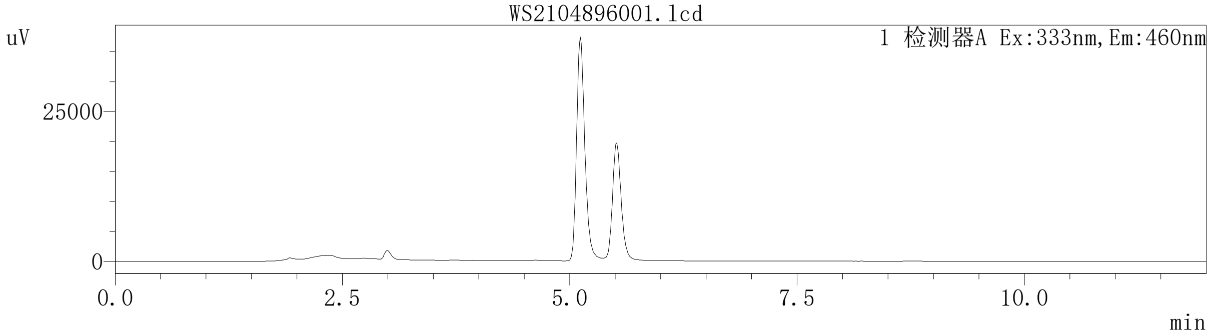
WS2104896001.lcd
uV
1 检测器A Ex:333nm,Em:460nm
25000
0
0.0
2.5
5.0
7.5
10.0
min

 检验者： 复核者： 复核日期：

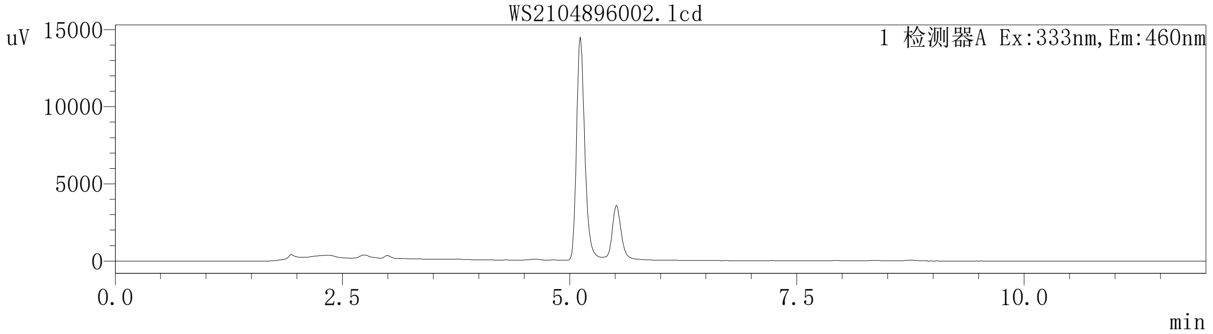

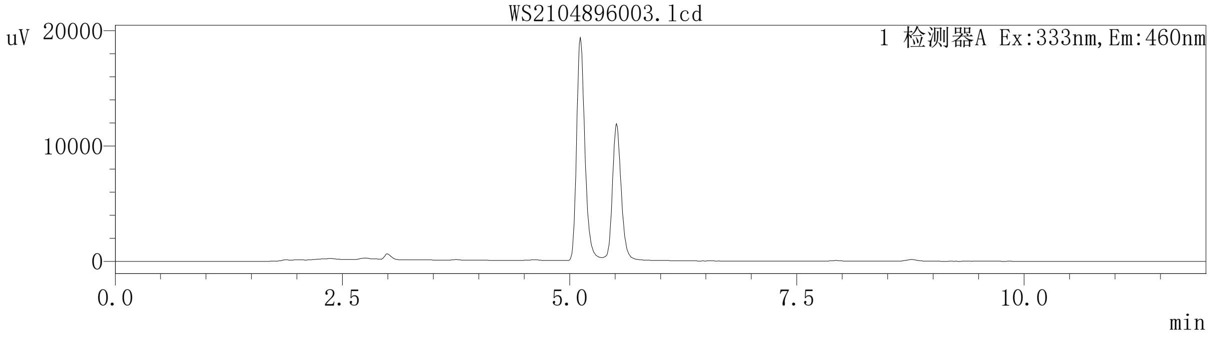

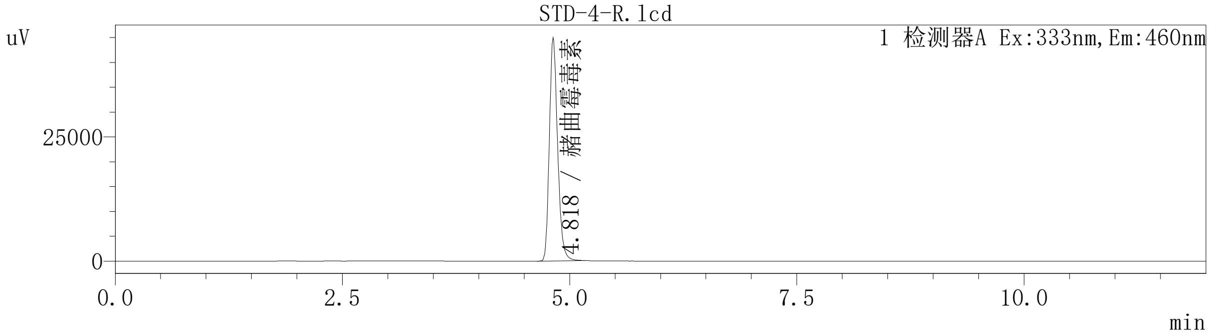

<< 检测器A >>
ID#1 化合物名: 赭曲霉毒素

标题	保留时间	面积	浓度	浓度单位	样品瓶号	分析日期	分析时间
试剂空白.lcd	0.000	0	0.0000	ng/ml	1	2021/9/2	12:44:33
STD-1.lcd	4.815	5310	0.1887	ng/ml	3	2021/9/2	12:57:07
STD-2.lcd	4.818	14228	0.5056	ng/ml	4	2021/9/2	13:09:40
STD-3.lcd	4.814	68494	2.4342	ng/ml	5	2021/9/2	13:22:13
STD-4.lcd	4.817	284548	10.1127	ng/ml	6	2021/9/2	13:34:47
STD-5.lcd	4.816	1372043	48.7619	ng/ml	7	2021/9/2	13:47:20
STD-6.lcd	4.819	2830906	100.6094	ng/ml	8	2021/9/2	13:59:54
过程空白.lcd	0.000	0	0.0000	ng/ml	2	2021/9/2	14:12:28
WS2104896001.lcd -TDs	0.000	0	0.0000	ng/ml	12	2021/9/2	15:02:44
WS2104896002.lcd -T. koningii FTDs	0.000	0	0.0000	ng/ml	13	2021/9/2	15:15:18
WS2104896003.lcd -A.niger FTDs	0.000	0	0.0000	ng/ml	14	2021/9/2	15:27:53
STD-4-R.lcd	4.818	283508	10.0758	ng/ml	6	2021/9/2	15:40:27

校准曲线

ID号　　　　　　　　　　　　: 1
化合物名　　　　　　　　　　: 赭曲霉毒素
定量计算法　　　　　　　　　: 外标法
校准曲线公式　　　　　　　　: f(x)=28137.6*x+0
相关系数(R)=0.9998883　拟合度(R^2)=0.9997765　剩余值的平方和(RSS)=1.521233e+009
平均 RF 2.776827e+004　RF标准偏差 7.694208e+002　RF相对标准偏差RSD 2.770863
校准曲线类型　　　　　　　　: 直线
原点　　　　　　　　　　　　: 通过
加权方法　　　　　　　　　　: 无
检测器名　　　　　　　　　　: 检测器A

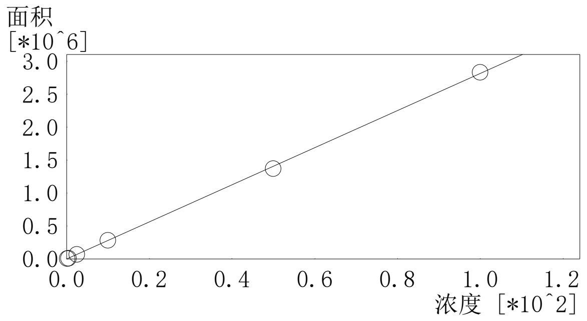

#	浓度(比率)	平均面积
1	0.2	5310
2	0.5	14228
3	2.5	68494
4	10	284548
5	50	1372043
6	100	2830906

 检验者：　　　　复核者：　　　　复核日期：
