# Supplementary material for: Solid-state fermentation by Aspergillus niger and Trichoderma koningii improves the quality of tea dregs for use as feed additives
Source: PLoS One. 2021 Nov 12;16(11):e0260045. doi: 10.1371/journal.pone.0260045 (PMC8589212; doi:10.1371/journal.pone.0260045)
Supplement: S1 Table — (DOCX) [file pone.0260045.s002.docx]

**S2 Table Criteria for odor quality assessment.**

| Odor | Sensory indicators | Scores |
| --- | --- | --- |
| Top | Pleasant and thicker tea fragrance | 4–5 |
| Good | Tea fragrance | 2–3 |
| General | Faint tea fragrance | 1–2 |
| Inferior | Decayed | 0–1 |
