# Supplementary material for: Solid-state fermentation by Aspergillus niger and Trichoderma koningii improves the quality of tea dregs for use as feed additives
Source: PLoS One. 2021 Nov 12;16(11):e0260045. doi: 10.1371/journal.pone.0260045 (PMC8589212; doi:10.1371/journal.pone.0260045)
Supplement: S2 Table — (DOCX) [file pone.0260045.s003.docx]

**S3 Table Odor scores from fermented tea dregs.**

| **Items** | Odor scores |
| --- | --- |
| **Control** | 2.38 ± 0.35^b^ |
| ***T.koningii*** | 4.38 ± 0.52^a^ |
| ***A.niger*** | 4.19 ± 0.59^a^ |
| ***P*-value** | <0.001 |

Means with different letters in the same row (a–c) indicated a significant difference according to Duncan’s multiple comparison tests at *P*< 0.05.
